# Molecular and physiological responses of two quinoa genotypes to drought stress

**DOI:** 10.3389/fgene.2024.1439046

**Published:** 2024-08-09

**Authors:** Xiaolin Zhu, Wenyu Liu, Baoqiang Wang, Ling Yang

**Affiliations:** ^1^ College of Life Science and Technology, Gansu Agricultural University, Lanzhou, China; ^2^ Gansu Provincial Key Laboratory of Aridland Crop Science, Gansu Agricultural University, Lanzhou, China; ^3^ Gansu Academy of Agricultural Sciences, Lanzhou, China; ^4^ School of Biological and Pharmaceutical Engineering, Lanzhou Jiaotong University, Lanzhou, China

**Keywords:** quinoa, drought stress, physiology, transcriptome, molecular mechanism

## Abstract

Quinoa is an important economic food crop. However, quinoa seedlings are susceptible to drought stress, and the molecular mechanism of drought tolerance remains unclear. In this study, we compared transcriptomic and physiological analyses of drought-tolerant (L1) and susceptible (HZ1) genotypes exposed to 20% PEG for 3 and 9 days at seedling stage. Compared with HZ1, drought stress had less damage to photosynthetic system, and the contents of SOD, POD and CAT were higher and the contents of H_2_O_2_ and O_2_^−^were lower in L1 leaves. Based on the RNA-seq method, we identified 2423, 11856, 1138 and 3903 (HZ1-C3-VS-T3, HZ1-C9-vs-T9, L1-C3-vs-T3 and L1-C9-vs-T9) annotated DEGs. Go enrichment was shown in terms of Biological Process: DEGs involved in biological processes such as metabolic process, cellular process, and single-organism process were most abundant in all four comparison treatments. In Molecular Function: the molecular functions of catalytic activity, binding and transporter activity have the most DEGs in all four processes. Cellular Component: membrane, membrane part, and cell have the most DEGs in each of the four processes. These DEGs include AP2/ERF, MYB, bHLH, b-ZIP, WRKY, HD-ZIP, NAC, C3h and MADS, which encode transcription factors. In addition, the MAPK pathway, starch and sucrose metabolism, phenylpropanoid biosynthesis and plant hormone signal transduction were significantly induced under drought stress, among them, *G-hydrolases-66*, *G-hydrolases-81*, *G-hydrolases-78*, *Su-synthase-02*, *Su-synthase-04*, *Su-synthase-06*, *BRI1-20* and *bHLH17* were all downregulated at two drought stress points in two genotypes, *PP2C01*, *PP2C03*, *PP2C05-PP2C07*, *PP2C10*, *F-box01* and *F-box02* were upregulated at two drought stress points in two genotypes. These results agree with the physiological responses and RNA-seq results. Collectively, these findings may lead to a better understanding of drought tolerance, and some of the important DEGs detected in this study could be targeted for future research. And our results will provide a comprehensive basis for the molecular network that mediates drought tolerance in quinoa seedlings and promote the breeding of drought-resistant quinoa varieties.

## Introduction

Drought is becoming more serious and widespread with the increasing global temperature, which affects 64% of the global land area (Lesk et al., 2016). Drought stress occurs when crops do not receive sufficient rain or irrigation water during the growing season. For example, rice has suffered significant yield losses of 18%–60% due to water scarcity (Bernier et al., 2007) and wheat has suffered yield losses of 10%–50% due to water scarcity (Amir et al., 2011). N agricultural production, drought stress emerges as a paramount limiting factor for crop growth, primarily due to its disruptive impact on the delicate carbon balance within plants. This balance, heavily reliant on photosynthesis, serves as the cornerstone for crop yield, with leaf photosynthesis specifically being the fundamental process underpinning the formation of harvestable produce (Zhu et al., 2010). Therefore, photosynthesis has been identified as a critical determinant of crop yield, and many studies have advocated improved photosynthesis breeding as a sustainable means of increasing crop yield (Liu et al., 2020). The reduction of leaf photosynthesis under drought stress is thought to be mainly caused by the significant reduction of quantum efficiency, maximum photochemical efficiency and carboxylation efficiency, leaf water potential level and chlorophyll content had significant effects on photosynthesis (Meinzer et al., 2017). Leaf water potential decreases under drought stress, resulting in stomatal closure, and if leaf water potential is kept at low levels for a long time, it can lead to carbon starvation and death in plants (Wang et al., 2018). Lack of CO_2_ will promote photooxidative stress in chloroplasts, resulting in decreased photochemical activity of PSII and the formation of ROS, the chlorophyll fluorescence parameters were also affected by drought stress, which generally showed the increase of F0 value and the decrease of Fv and Fm value. In addition, the results showed that Pn, Tr and Gs of leaves were significantly decreased, while intercellular CO_2_ concentration was increased under drought stress (Zhang et al., 2014). Plant drought stress is physiologically complex, including osmotic stress and specific ionic toxicity, drought stress in plants is associated with nutrient imbalances, reduced cell division and expansion, and excessive production of ROS, which can trigger a cascade of oxidative reactions, this leads to inactivation of the enzyme and increase in Lipid peroxidation, the final product of which is MDA, which can be quantified as a marker of oxidative damage (Forni et al., 2017). ROS produced under drought stress will be eliminated by boosting the activity of enzymes in the antioxidant system, including SOD, POD and CAT. Therefore, understanding drought-tolerant mechanisms of crops and developing drought-tolerant varieties are essential to maintain crop yields under drought conditions.

RNA sequencing offers a powerful tool for delving deeper into the transcripts of all active genes and their intricate splicing variants within plants, enabling a comprehensive understanding of their expression patterns and functional diversity. Although RNA sequencing methods have revealed previously annotated differentially expressed genes, they have also contributed to the discovery of new transcripts (Liu et al., 2015; Braynen et al., 2017; Haider et al., 2017). At present, NAC, WRKY and BHLH transcription factors involved in regulating drought response have been identified in the transcriptome of alfalfa (*Medicago sativa* L.) (Wan et al., 2022). TAS14, an ABA-induced dehydrant protein, was identified as a molecular sensor for drought in the potato drought transcriptome, and this gene was found to be associated with drought recovery potential (Van Muijen et al., 2016). Consequently, transcriptome analysis can be employed to uncover the molecular features underlying drought response. Unfortunately, more detailed studies on the changes of transcription levels under drought stress in quinoa are not enough. Transcriptome analysis can be employed to uncover the molecular features underlying drought response.

Quinoa (*Chenopodium quinoa* L.) is an annual dicotyledonous self-pollinated plant of quinoa has high nutritional value and is called “Nutritional gold” by international nutritionists. The Food and Agriculture Organization of the United Nations believes that quinoa is the only plant-based food that can satisfy the basic nutritional needs of the human body, and officially recommends quinoa as the perfect all-nutrient food for humans. Quinoa boasts an impressive array of resilience characteristics, including cold-tolerance, drought-resistance, barren-hardiness, salt-tolerance, and various other abilities that enable it to thrive in diverse and often challenging environments. Therefore, it is of great significance to study the mechanism of quinoa drought resistance and identify the key genes related to drought tolerance, enabling quinoa to adapt to severe water shortages and mitigate economic losses, particularly in regions where quinoa cultivation is prevalent. However, so far, studies on drought resistance in quinoa have primarily focused on physiological changes; the underlying molecular mechanisms of drought tolerance remain poorly understood. In this study, we selected the drought-tolerant variety “Longly 1” and the drought-sensitive variety HZ for further investigation, aiming to elucidate the biochemical and molecular differences associated with their varying sensitivities to drought. To simulate drought stress, we treated the quinoa plants with 20% PEG-6000 and collected leaves at 3 and 9 days post-treatment, respectively, for the construction of cDNA libraries for sequencing on an Illumina platform, and analyze the differences in response to the gene expression profiling of drought over time. Functional annotation of differentially expressed genes (DEGs) was performed to identify potential candidate genes for drought resistance. This study provides the gene expression profiling data of quinoa leaves under drought stress, which will help to further analyze the molecular mechanism of quinoa drought resistance, to enrich plant genetic resources and lay a foundation for molecular level of drought-resistant quinoa.

## Materials and methods

The seeds of quinoa, including the drought-tolerant genotype Longli 1 and the drought-sensitive genotype HZ1, came from Gansu Academy of Agricultural Sciences. The seeds of quinoa, all of which were plump and of the same size, were first disinfected with 5% NaClO for 5 min, and then washed with sterile distilled water five times. The seeds were then sown in 10-cm diameter round flower pots (10 plants per pot) with a 1:1 ratio of vermiculite to nutrient soil and all other growing conditions were consistent. The experiment was conducted in a Gansu Agricultural University greenhouse. The mean diurnal temperature and relative humidity were set at 28/20°C ± 2°C and 60/55% the light is 16 h/8 h (day/night), respectively. Stress treatment was carried out at the age of 2 months, with 60 pots per treatment. Control group: normal watering; treatment group: 20% PEG simulated drought stress; after treatment 0,3,6, 9 and 12 days, at 8:00–10:00 a.m., the leaves of quinoa were taken and immediately frozen in liquid nitrogen, stored at −80°C. The seeds of quinoa, all of which were plump and of the same size, were first disinfected with 5% NaClO for 5 min, and then washed with sterile distilled water five times.

### Determination of photosynthetic characteristics related indicators

The photosynthetic parameters and fluorescence parameters of 9 quinoa seedlings were measured at 0,3,6,9 and 12 days after treatment, the second and 3rd leaves of the same size, without diseases and insect pests, and without damage were selected from the leaves of quinoa. PN, CI, GS and TR, were measured by Li-6400XT portable photosynthetic instrument of LI-COR company. The leaf chamber temperature was set at 30°C, the light intensity was 1,200 μmol·m^−2^·s1, and the flow rate was 450 μmol·mol^−1^. Fluorescence parameters: the quinoa leaves were dark for 30 min, then the maximum photochemical efficiency (Fv/Fm) of PSII was measured by fluorometer. Chlorophyll was measured by chlorophyll meter.

### Determination of physiological indexes

The activity of POD was determined by guaiacol method (Liu et al., 2014). SOD was determined by NBT reduction method (Li et al., 2000). CAT was determined by Abei method (Abei, 1984). The content of hydrogen peroxide (H_2_O_2_) was determined by KI chromogenic method (Willekens et al., 1997), the concentration of hydroxyl radical (·OH) was determined by 2-deoxy-d-ribose chromogenic method (Liu et al., 2009), and the production rate of superoxide anion radical (O_2_•-) was determined by 4-aminobenzenesulfonic acid method (Elstner and Heupel, 1976). The experiment consisted of three biological and technical replicates.

### Total RNA extraction, cDNA library construction and transcriptome sequencing

Total RNA was extracted by Trizol reagent (Invitrogen, Carlsbad, CA, USA). Nanodrop 2000 (Thermo Fisher Scientific, Waltham, MA, USA) was used to determine the concentration and purity of RNA. The integrity of RNA was detected by 1% agarose gel electrophoresis. The integrity of the RNA was further scrutinized utilizing an Agilent 2,100 bioanalyzer (Agilent Technologies, Santa Clara, CA, USA). The mRNA extraction quality and concentration of all samples were detected; mRNA is rich in Oligo (dT) magnetic beads. In addition, the fragmentation buffer is added to the mRNA and cut into short fragments. Using mRNA as a template, cDNA was reverse transcribed using six-base random primers. The double-stranded cDNA sample was purified, end-repaired, added with a poly A) tail, and ligated to a sequence adaptor to create a 24-sample cDNA library. All cDNA libraries were then sequenced on the Illumina HiSeqTM 2,500 platform (Illumina, San Diego, CA, USA).

### Data analysis, assembly and annotation

The original data is first processed by an internal Perl script and clean data is obtained. Meanwhile, we calculated the Q20, Q30 values, GC content, and sequence repeat level of the clean data. All downstream analyses are based on high quality clean data. These sequencing analysis procedures are performed as in the literature (Grabherr et al., 2011; Chen et al., 2019). According to NR gene function annotation; Pfam; kOG/COG/eggNOG (Tatusov et al., 2000; Koonin et al., 2004); Swiss-Prot (Apweiler et al., 2004); KEGG (Kanehisa et al., 2004); and GO (Ashburner et al., 2000) databases are used to analyze other content.

### Construction of protein interaction networks

Using Cytoscape software (Shannon et al., 2003), the protein interaction network was constructed based on the protein sequence.

### Quantification and difference analysis of gene expression level

Gene expression levels were calculated by RSEM (Dewey and Li, 2011). Differential expression analysis was performed using the DESeq R package (1.10.1) (Anders and Huber, 2012). The adjusted *p*-value found |log_2_Fold Change| > = 1, and FDR <0.05. The FPKM values and the overall distribution of PCC were calculated using R (www.r-project.org) and are represented as graphs and heat maps, respectively. We used KOBAS (Xie et al., 2011) software to test the statistical enrichment of differentially expressed genes in the KEGG pathway (Kanehisa and Goto, 2000).

## Results

### Analysis of photosynthetic parameters

**FIGURE 1:**
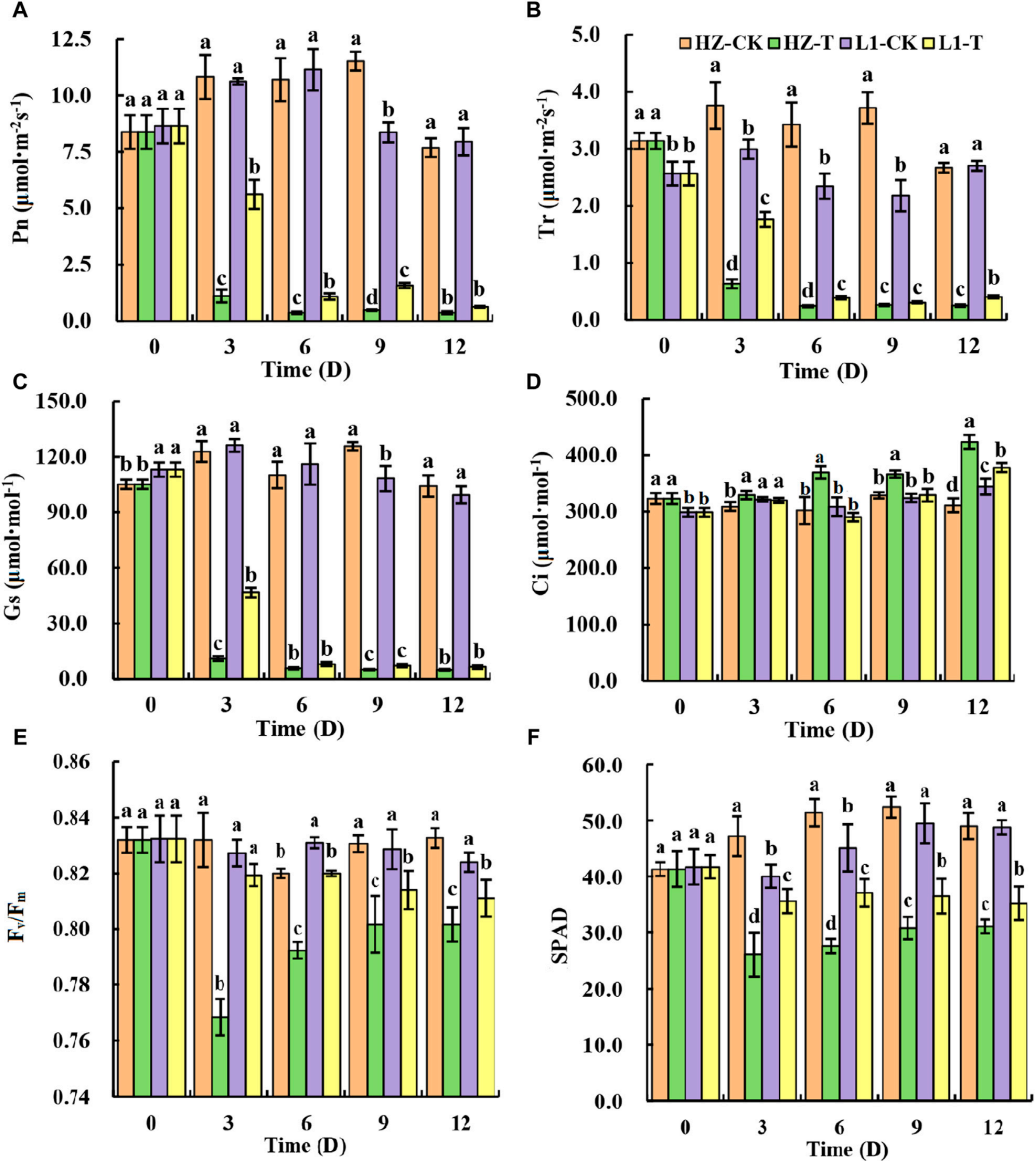
Effects of 20% PEG stress on photosynthetic parameters of different drought-resistant genotypes. Note: Different lowercase letters in the graph indicate that there are significant differences between different varieties under the same stress water potential (*p* < 0.05).

Through the previous seed germination experiment, photosynthetic parameters were analyzed at different time points after applying 20% PEG stress to two materials: L1, a drought-tolerant material, and HZ1, a drought-sensitive material. Studies have shown that drought stress will adversely affect the carbon assimilation capacity of crops, thereby reducing the photosynthetic rate. In this study, it can be seen from Figures 1A–F that 20% PEG drought stress greatly reduced the Pn, Gs and Tr of the two materials. For HZ1, after 20% PEG stress, the Pn and Tr value continued to decrease within 0–6 days, and the Pn value decreased by 89.66%, 96.49%, 95.68% and 95.11% respectively compared with the control at 3,6,9 and 12 days after 20% PEG stress. The Tr value decreased by 83.16%, 92.94%, 92.91% and 90.57% respectively compared with the control at 3,6,9 and 12 days after 20% PEG stress. The Gs value showed a continuous decreasing trend within 0–12 days, and the Gs value decreased by 90.99%, 94.82%, 95.97% and 95.23% at 3,6,9 and 12 days after 20% PEG stress, respectively. The Ci increased continuously in HZ1 material during 20% PEG drought stress, which increased by 6.68%, 22.4%, 11.39% and 36.19% respectively compared with the control. For L1, after exposure to 20% PEG stress, both its Pn value and Tr value exhibited a continuous decrease over the period of 0–6 days; the Pn value decreased by 47.08%, 90.16%, 81.11% and 92.04% respectively compared with the control at 3,6,9 and 12 days after 20% PEG stress. The Tr value decreased by 41.12%, 83.29%, 85.87% and 84.95% respectively compared with the control at 3,6,9 and 12 days after 20% PEG stress. The Gs value showed a continuous decreasing trend within 0–12 days, and the Gs value decreased by 63.04%, 93.07%, 93.35% and 93.47% respectively at 3,6,9 and 12 days after 20% PEG stress. Ci showed a trend of increasing first, then decreasing and then increasing in L1 material during 20% PEG drought stress, and there was no significant difference between the value and the control on the 3rd and 9th day of drought stress. In this study, we analyzed Fv/Fm and SPAD under 20% PEG stress. It can be seen from Figures 1E, F that Fv/Fm and SPAD values decreased significantly after drought stress. Fv/Fm decreased by 7.65%, 3.37%, 3.49% and 3.72% respectively compared with the control, and SPAD decreased by 44.91%, 46.49%, 41.41% and 36.73% respectively compared with the control. Fv/Fm decreased most obviously on the third day. After 20% PEG stress, SPAD values were significantly lower than the control at each time point, decreasing by 11.25%, 17.96%, 26.46%, and 28.07%, respectively. Similarly, we observed a significantly larger decrease in both Fv/Fm and SPAD values in HZ1 compared to that in L1. In general, 20% PEG stress caused serious damage to the photosynthetic system of the two materials, especially for HZ1 material.

### Effects of PEG stress on antioxidant enzyme activities and active oxygen accumulation in leaves of quinoa

**FIGURE 2:**
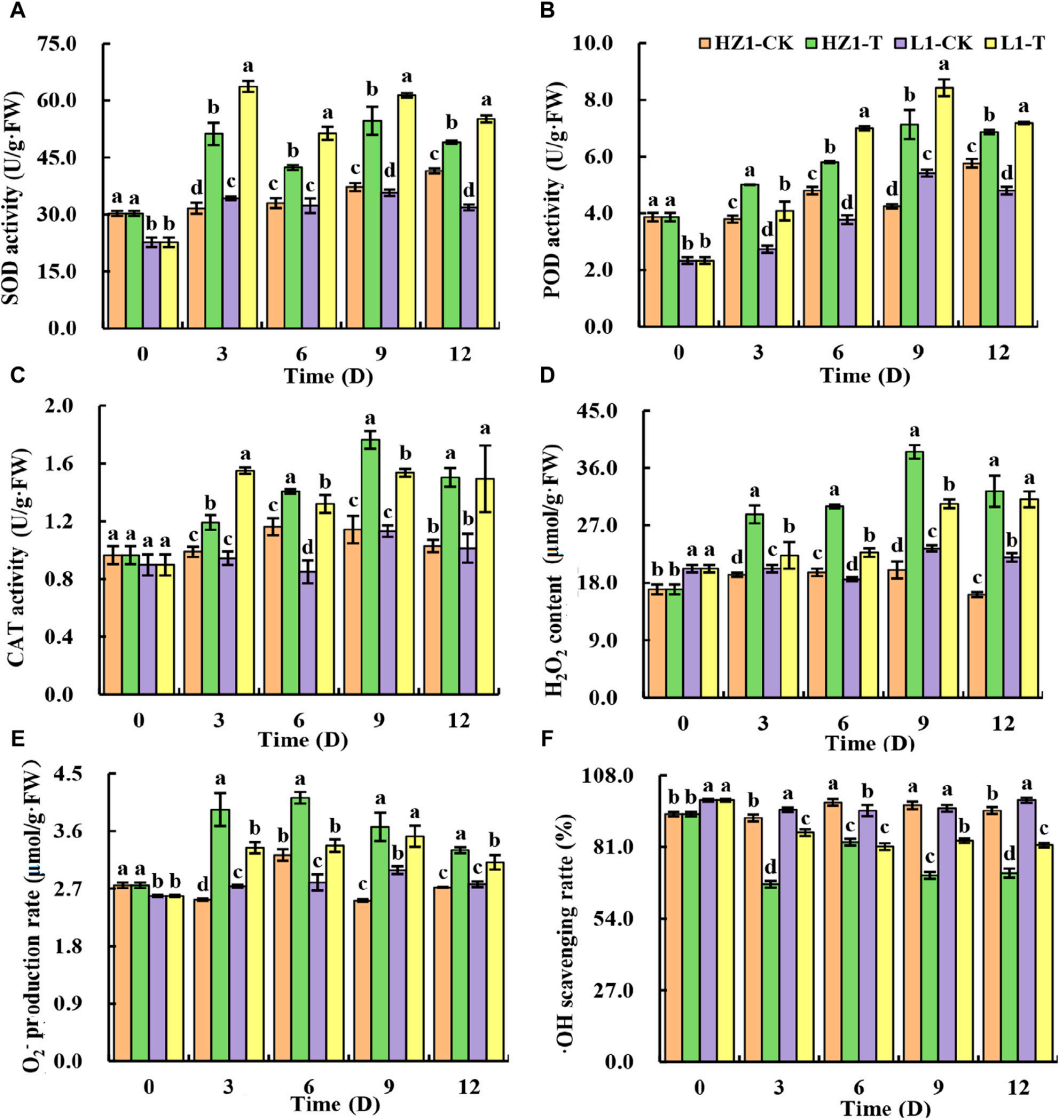
Effects of 20% PEG stress on antioxidant enzyme activity and free radical content in different drought-resistant genotypes. Note: Different lowercase letters in the figure indicate significant differences between different varieties under the same stress water potential (*p <* 0.05).

To further elucidate the mechanism of quinoa drought tolerance, we investigated the contents of antioxidant enzyme activities and reactive oxygen species in quinoa leaves under 20% PEG stress (Figures 2A–F). In HZ1, we found that drought stress significantly increased the SOD, POD and CAT activity of quinoa leaves. The SOD activity increased by 61.80%, 28.38%, 46.92% and 18.13% at 3,6,9 and 12 days after stress, respectively. The POD activity increased by 32.08%, 21.01%, 67.84% and 19.07%, respectively, and the CAT activity increased by 20.57%, 21.09%, 54.38% and 46.19%, respectively. In L1, we found similar results to those in HZ1 The SOD activity increased by 85.87%, 58.97%, 71.84% and 73.20% at 3,6,9 and 12 days after stress, respectively. The POD activity increased by 49.39%, 85.43%, 55.54% and 49.65%, respectively, and the CAT activity increased by 64.31%, 55.29%, 35.76% and 47.37%, respectively. Thus, the elevated levels of antioxidant enzyme activity constitute one of the key factors contributing to L1’s drought tolerance. In addition, we studied the accumulation of ROS under drought stress, and found that after drought stress, the production rate of O_2_-in the two materials increased first and then decreased with the prolongation of drought stress. Compared with the control, the production rate of O_2_-in HZ1 increased by 55.76%, 27.69%, 45.89% and 21.39%, respectively, at 3,6,9 and 12 days after stress. Compared with the control, the production rate of O_2_-in L1 increased by 22.02%, 20.55%, 17.70% and 12.41%, respectively, compared with the control. We found that O_2_- production rate in HZ1 was significantly higher than that in L1 at different time points after stress. As far as H_2_O_2_ was concerned, with the increase of stress days, the content of H_2_O_2_ in quinoa leaves increased first and then decreased in HZ1, and its content reached the maximum on the 9th day, which was 92.469% higher than that of the control, while it showed a continuous increase in L1. Under drought stress, the OH scavenging rate decreased in both materials, but the decrease observed in HZ1 was greater than that in L1. The OH scavenging rate in HZ1 decreased by 27.19%, 15.34%, 27.30% and 24.93% compared with the control at 3,6,9 and 12 days after stress, respectively. In L1, it decreased by 9.06%, 14.37%, 12.79% and 17.18%, respectively.

### RNA sequencing analysis

At the six-leaf stage, quinoa leaves treated with drought for 3 days and 9 days were collected for later transcriptomic analysis. Drought-tolerant material Longly 1, 12 samples (L1-C3-1, L1-C3-2, L1-C3-3, L1-C9-1, L1-C9-2, L1-C9-3, L1-T3-1, L1-T3-2, L1-T3-3, L1-T9-1, L1-T9-2, L1-T9-3), and sensitive material HZ1. 12 materials (HZ1-C3-1, HZ1-C3-2, HZ1-C3-3, HZ1-C9-1, HZ1-C9-2, HZ1-C9-3, HZ1-T3-1, HZ1-T3-2, HZ1-T3-3, HZ1-T9-1, HZ1-T9-2, HZ1-T9-3), a total of 24 samples. 24 samples were used to make cDNA libraries. The original data is stored in the NCBI sequence read archive, and the SRA number is: SUB12488150. Data (Supplementary Table S1) after removing the low-quality sequences and adaptor sequences, a total of 155.95 Gb Clean Data was obtained. The percentage of Q20 bases was at least 97.01%, and the percentage of Q30 bases was at least 92.24%. The GC content of 24 samples was above 43.81%. The Clean Reads of each sample were compared with the designated reference genome, and the comparison efficiency ranged from 86.50% to 96.67%. Mortazavi used the per million mapping reading (FPKM) method to measure the transcript abundance of each gene. In order to check the repeatability and reliability of the experimental results, we used Pearson correlation test to test the expression pattern relationship between drought treatment T) and control C) (Supplementary Figure S1). The RNA-seq correlation coefficient of FPKM was between the repetition of drought treatment and control, indicating that the gene expression patterns were similar, indicating the repeatability and reliability of the experimental results. In addition, in order to analyze the similarities and differences between 24 samples, principal component analysis (PCA) was performed on 24 samples. The results showed that the sensitive and resistant materials were clustered together in different treatment replicates (Supplementary Figure S2). These findings demonstrate the reproducibility and reliability of the experiment.

### RNA sequencing analysis and identification of differentially expressed genes

**FIGURE 3:**
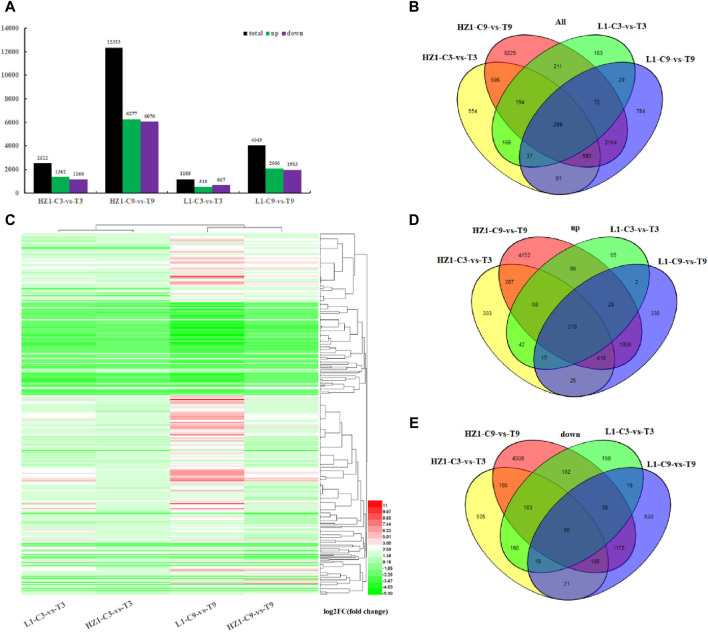
Differentially expressed genes in quinoa materials HZ-1 and L1 under drought stress. Note: **(A)** Number of differentially expressed genes in HZ-1 and L1 after 3 and 9 days of drought stress. **(B)** Venn diagram of differentially expressed genes. **(C)** Cluster analysis heatmap of 299 common drought response genes. **(D)** Venn diagram of upregulated genes in HZ-1 and L1 after 3 and 9 days of drought stress. The Venn diagram of downregulated genes in HZ-1 and L1 after 3 days and 9 days of drought stress.

After 3 and 9 days of drought treatment, the differences in gene expression between the two materials were assessed utilizing the Cuffdiff software package. Generally, Fold Change ≥2 and FDR ≤0.01 are defined as differentially expressed genes (Figures 3A–E). On the third day after drought stress, 2,522 DEGs were obtained in drought-sensitive material HZ1. In drought-tolerant material L1, 1,185 DEGs were obtained. In sensitive materials, the number of upregulated genes exceeded that of downregulated genes, whereas in resistant materials, the reverse trend was observed. On the 9th day after drought stress, 12,353 differentially expressed genes were obtained in HZ1. In L1, 4,049 differentially expressed genes were obtained. At the same time, we found that with the prolongation of drought stress, the differentially expressed genes in response to drought also increased, and the differentially expressed genes in sensitive materials were always more than those in resistant materials.

### Annotation and difference analysis of differentially expressed genes

In this study, the obtained DEGs were functionally annotated using the reference genome (Chenopodium-quinoa. PI- 614886. Genome. fa), of which 2,423 (96.07%), 11,856 (95.97%), 1,138 (96.03%) and 3,903 (96.39%) genes under HZ1-C3-vs-T3, HZ1-C9-vs-T9, L1-C3-vs-T3 and L1-C9-vs-T9 were annotated, respectively (Table 1).

**TABLE 1:** Basic annotation information.

	Treatment group	Total annotation differential genes	Upregulation annotated genes	Downregulation annotated genes
HZ1	HZ1-C3-vs-T3	2423	1329	1094
	HZ1-C9-vs-T9	11856	6138	5718
L1	L1-C3-vs-T3	1138	511	627
	L1-C9-vs-T9	3903	1990	1913

### KEGG and GO enrichment analysis of differentially expressed genes

**FIGURE 4:**
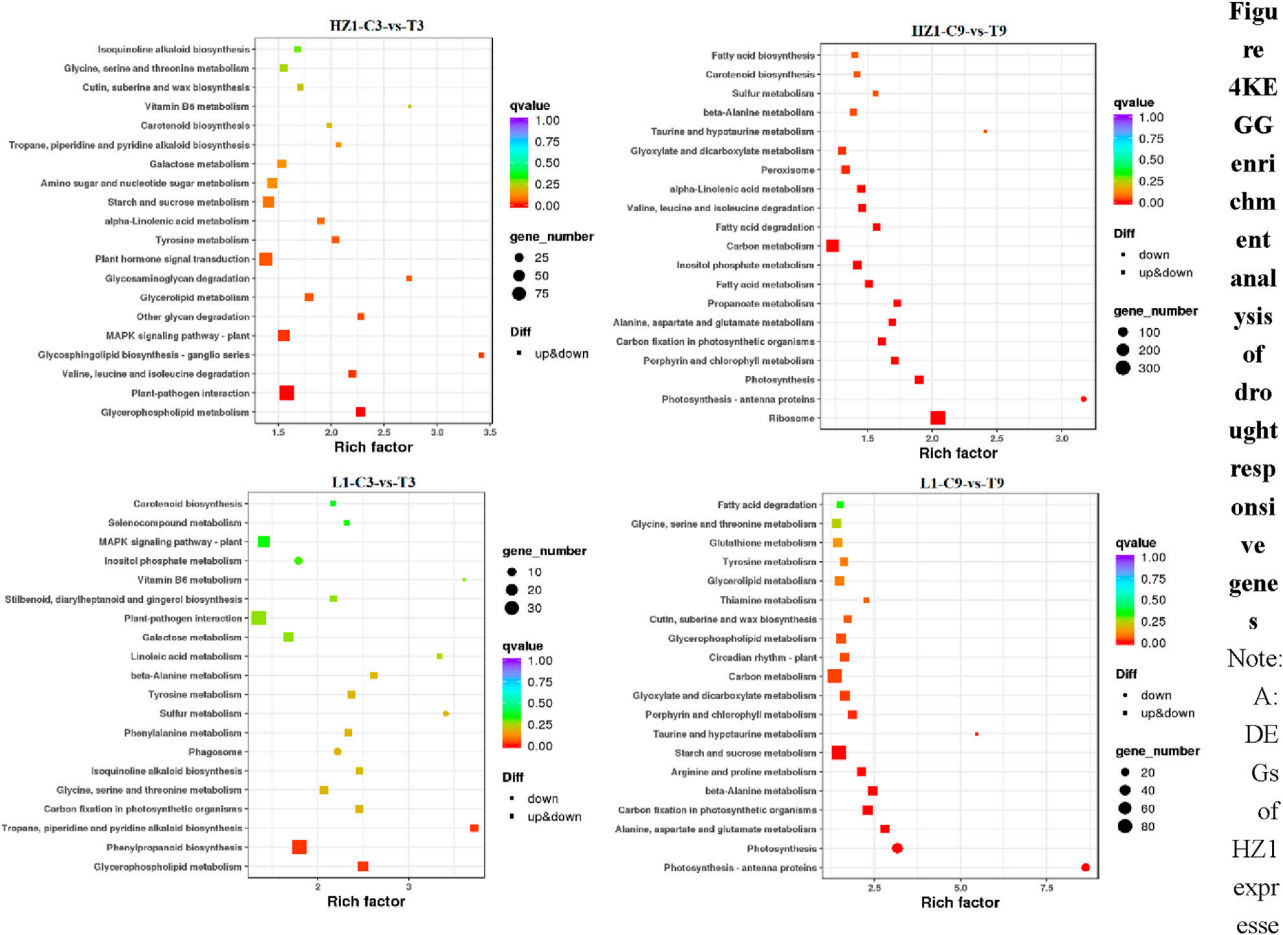
KEGG enrichment analysis of drought responsive genes. Note: DEGs of HZ1 expressed under 3D drought stress. DEGs of HZ1 expressed under 9D drought stress. DEGs expressed by L1 under 3D drought stress. DEGs expressed by L1 under 9D drought stress. The drought treatment was marked as control C and treatment (T).

In this study, we performed KEGG enrichment analysis on 2,423, 11,856, 1,138 and 3,903 differentially expressed genes, and the top 20 enrichment pathways are shown in the figure below (Figure 4). Drought stress day 3, there were many differential genes enriched in Plant-pathogen interaction (92), Plant hormone signal transduction (71), plant MAPK signaling pathway (53) in HZ1. There were many differential genes enriched in the pathways of Phenylpropanoids biosynthesis (36), Plant-pathogen interaction (36), plant MAPK signaling pathway (22) in L1. On the 9th day of drought stress, there were more differential genes enriched in Ribosome (333), Carbon metabolism (230), Inositol phosphate metabolism (90) in HZ1. There were many differentially expressed genes enriched in the pathways of Starch and sucrose metabolism (89), Carbon metabolism (83), Photosynthesis (44) in L1.

**FIGURE 5:**
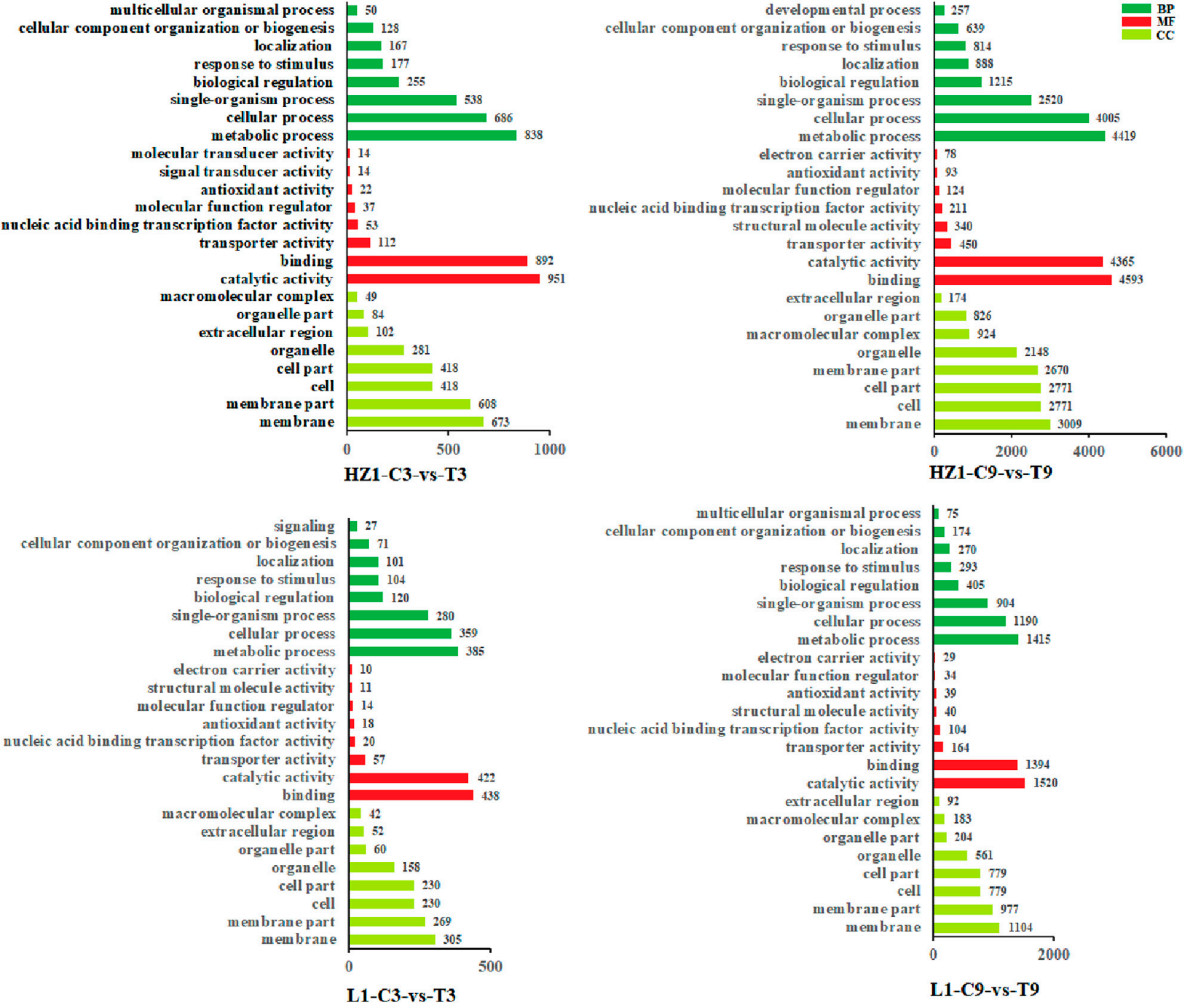
Gene ontology enrichment analysis of drought response genes. Note: DEGs of HZ1 expressed under 3D drought stress. DEGs of HZ1 expressed under 9D drought stress. DEGs expressed by L1 under 3D drought stress. DEGs expressed by L1 under 9D drought stress. The GO terms shown here are the highest biological process (BP), molecular function (MF), and cellular component (CC) categories from tolerant (L1) and susceptible (HZ1). The drought treatment was marked as control (C) and treatment (T).

In this study, we performed gene ontology (GO) enrichment analysis on 2,423, 11,856, 1,138 and 3,903 DEGs (Figure 5). GO enrichment analysis can divide these DEGs into three areas: biological process (BP), molecular function (MF) and cellular component (CC). On the third day of drought stress, there were 1896 differential genes involved in these three fields in HZ1-C3-vs-T3, involving 47 GO terms, and 899 differential genes involved in these three fields in L1-C3-vs-T3, involving 43 GO terms. On the 9th day of drought stress, there were 9,630 differential genes involved in these three fields in HZ1-C9-vs-T9, involving 50 GO terms, and 899 differential genes involved in these three fields in L1-C9-vs-T9, involving 50 GO terms. In addition, we mapped the first eight aspects of these processes as shown in the figure below. On the 3rd and 9th day of drought stress, in terms of BP: DEGs involved in metabolic process, cellular process and single-organism process were the most in the four comparison treatments. In terms of MF: catalytic activity, binding and transporter activity have the most DEGs under the four treatments. In terms of CC: membrane, membrane part and cell composition had the most DEGs under the four treatments.

### Transcription factor analysis of differential expression under drought stress

**FIGURE 6:**
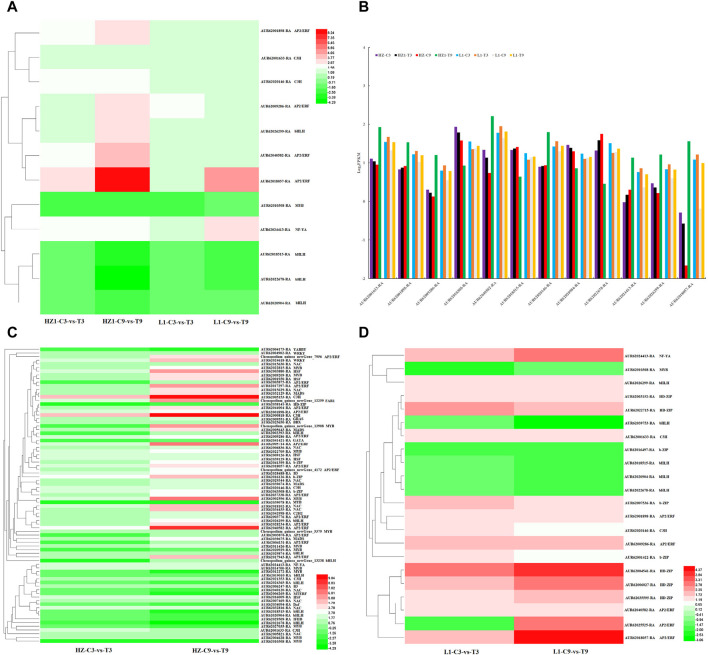
Analysis of differentially expressed transcription factors in leaves under 20% PEG simulated drought stress. Note: **(A)** 12 transcription factors shared by HZ1-C3-vs-T3, HZ1-C9-vs-T9, L1-C3-vs-T3 and L1-C9-vs-T9. **(B)** Expression of 12 common transcription factors based on Log_2_FPKM. **(C,D)**: Transcription factors co-expressed in HZ1 and L1 genotype materials on day 3 and 9 of drought stress.

In addition, we analyzed the DEGs encoding transcription factors, and identified 60 (35 upregulated, 25 downregulated), 157 (112 upregulated, 45 downregulated), 228 (116 upregulated, 112 downregulated) and 660 (375 upregulated, 286 downregulated) DEGs in HZ1-C3-vs-T3, HZ1-C9-vs-T9, L1-C3-vs-T3 and L1-C9-vs-T9, respectively. These DEGs encoding TFs mainly include AP2/ERF, MYB, bHLH, b-ZIP, WRKY, HD-ZIP, NAC, C3H and MADS. Among these transcription factor families, 12 genes encoding transcription factors were differentially expressed in 4 paired comparisons (Figures 6A–D), including 4 AP2/ERF transcription factors (*AUR62001898-RA*, *AUR62009286-RA*, *AUR62018057-RA* and *AUR62040582-RA*). 2 C3H transcription factors (*AUR62001633-RA* and *AUR62020146-RA*), 4 BHLH transcription factors (*AUR62018515-RA*, *AUR62020904-RA*, *AUR62022678-RA* and *AUR62026299-RA*), 1 MYB transcription factor (*AUR62010308-RA*) and 1 NF-YA transcription factor (*AUR62024413-RA*). To further understand the changes in 12 common differentially expressed genes, log_2_FPKM values of these 12 differentially expressed transcription factors were used to construct a trend plot. We found that on the third day of drought stress, the expression levels of 12 genes in HZ1 and L1 had little difference between control and treatment. On the 9th day of drought stress, we found that there were significant differences in the expression of 12 genes between the treatment and the control in HZ1, while only AUR62040582-RA gene was found in LI. In addition, we found 81 differentially co-expressed transcription factor genes (Figure 6C) in the comparison between HZ1-C3-vs-T3 and HZ1-C9-vs-T9, which involved more transcription factors MYB (14), NAC (10), BHLH 9), AP2/ERF (16). On the third day of drought stress, the differential fold of 79 DEGs was significantly downregulated (except AUR62005153-RA and AUR62000818-RA), indicating that drought stress inhibited the expression of these genes in the short term; On the 9th day of drought stress, 21 differentially expressed transcription factors were significantly upregulated, indicating that long-term drought stress significantly induced the expression of these differentially expressed genes. In the comparison between L1-C3-vs-T3 and L1-C9-vs-T9, 22 differentially co-expressed transcription factor genes were found, among which HD-ZIP 5), b-ZIP 3), BHLH 5), AP2/ERF 5) were more involved. 15 differentially expressed genes encoding transcription factors were significantly upregulated (except for *AUR62010308-RA*, *AUR62039753-RA*, *AUR62016497-RA*, *AUR62018515-RA*, *AUR62020904-RA* and *AUR62022678-RA*). *AUR62025525-RA* was significantly downregulated on day 3 of drought stress and significantly upregulated on day 9. These results indicate that drought stress triggers the expression of a majority of transcription factors, with NAC, MYB, HD-ZIP, BHLH, and AP2/ERF being particularly closely associated with drought stress.

### Analysis of AP2/ERF transcription factors involved in response of quinoa to drought stress

**FIGURE 7:**
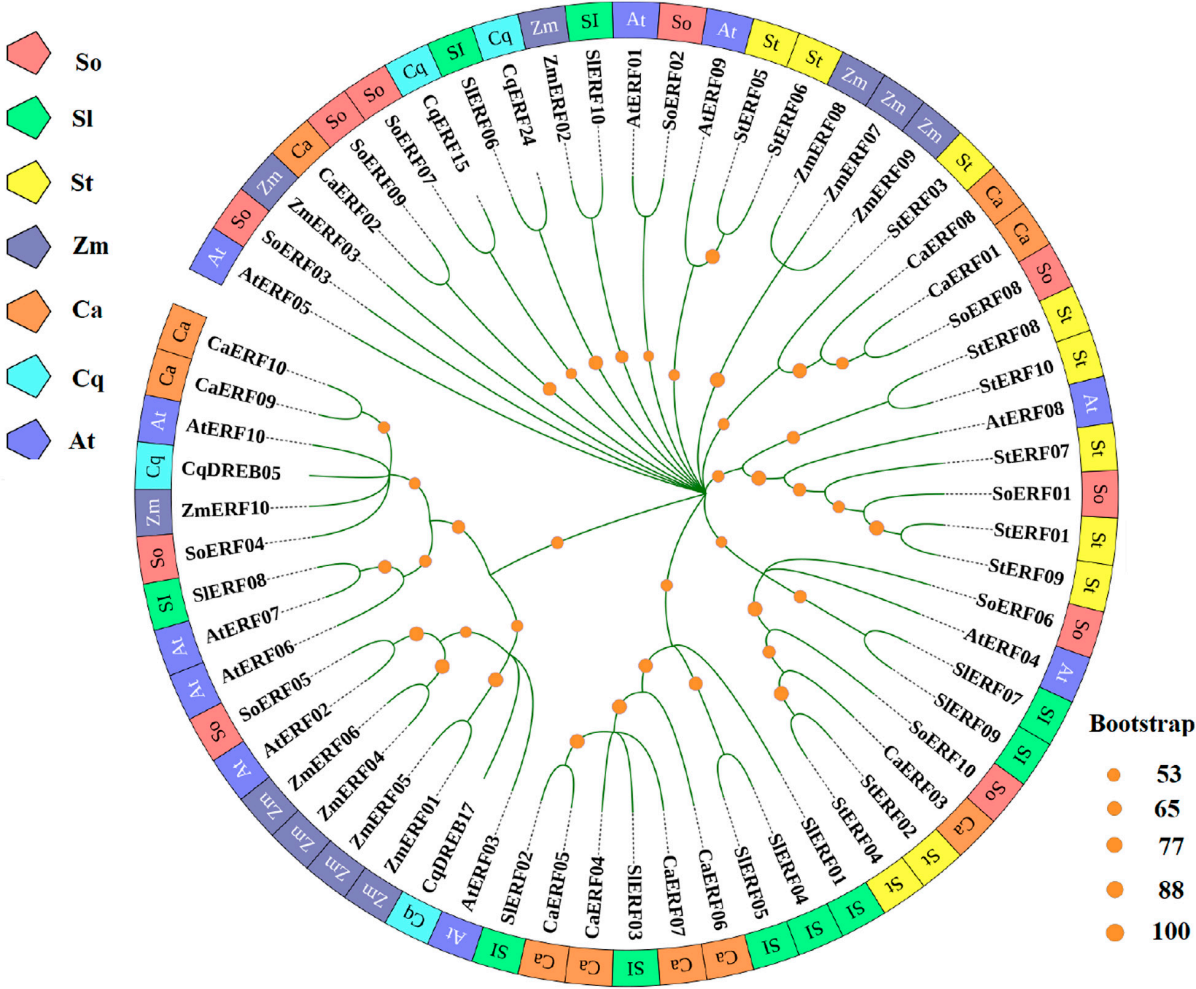
Shows the different sources of ERF transcription factors used to construct the phylogeny.

AP2/ERF (APETALA2/ethylene-responsive element binding factors) transcription factors are important regulators in plants, involved in plant morphogenesis, response to various stresses, hormone signal transduction and metabolite biosynthesis. Among them, ERF constitutes a subfamily within the AP2/ERF superfamily and plays a pivotal role in plant response to drought stress. In this study, we found that four genes encoding ERF transcription factors (*AUR62001898-RA*, *AUR62009286-RA*, *AUR62018057-RA* and *AUR62040582-RA*) were upregulated in HZ1-C3-vs-T3, HZ1-C9-vs-T9, L1-C3-vs-T3 and L1-C9-vs-T9. In order to further investigate the evolutionary relationship of ERF involved in drought stress responses in quinoa, we constructed a phylogenetic tree using ERF transcription factors from spinach, Arabidopsis, maize, tomato, pepper and potato (Figure 7). The results showed that CqDREB05 was closely related to AtERF10, CqDREB17 was closely related to AtERF03, CqERF15 was closely related to SoERF07, and CqERF24 was closely related to SlERF06. These findings serve as a solid foundation for the subsequent cloning and functional validation of CqERF genes.

### Identification of core drought-related genes by WGCNA analysis

**FIGURE 8:**
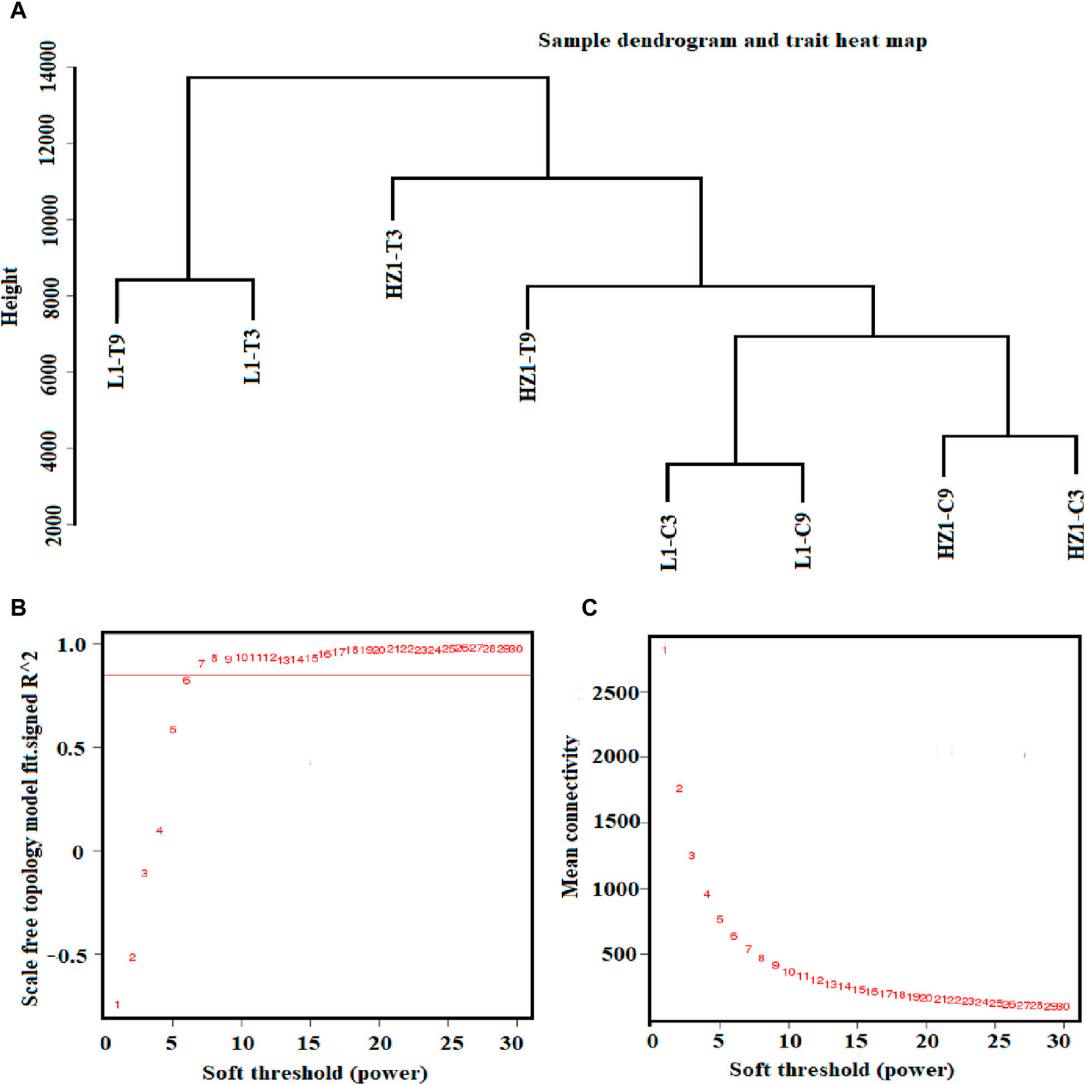
Sample hierarchical clustering tree and power value curve. Note: **(A)** The 8-sample hierarchical cluster graph is formed by combining the 3 repeated samples under each treatment; **(B)** The horizontal coordinate represents the power value, the vertical coordinate represents the correlation coefficient, and the red horizontal line represents the correlation coefficient of 0.9. **(C)** The horizontal coordinate represents the power value, and the vertical coordinate represents the average connectivity of genes.

Weighted Gene Co-expression Network Analysis (WGCNA) can classify genes with similar expression patterns into modules, thereby facilitating the exploration of genes with homogeneity through modular analysis methods. This approach can more effectively investigate similar genes and, when combined with prior physiological data, can accurately identify gene modules associated with specific traits. In WGCNA analysis, hierarchical clustering of samples is first performed using the levels of differentially expressed genes to observe the presence of any outlier samples. As shown in Figures 8A–C, in this study, samples with similar expression patterns were grouped into the same cluster without identifying any outliers. Additionally, the treatment and control of different materials were clustered separately, indicating that the gene expression patterns exhibit regularity, and samples with similar expression patterns tend to aggregate together.

In the choice of Power, this study selected a Power value of 7 under a correlation coefficient of 0.93 as the basis for subsequent WGCNA analysis (Figure 8). In addition, the change in the average gene connectivity under the Power value was analyzed. When the Power value reached 7, the average connectivity remained stable (Figure 8C), thereby fulfilling the prerequisites for subsequent analysis.

**FIGURE 9:**
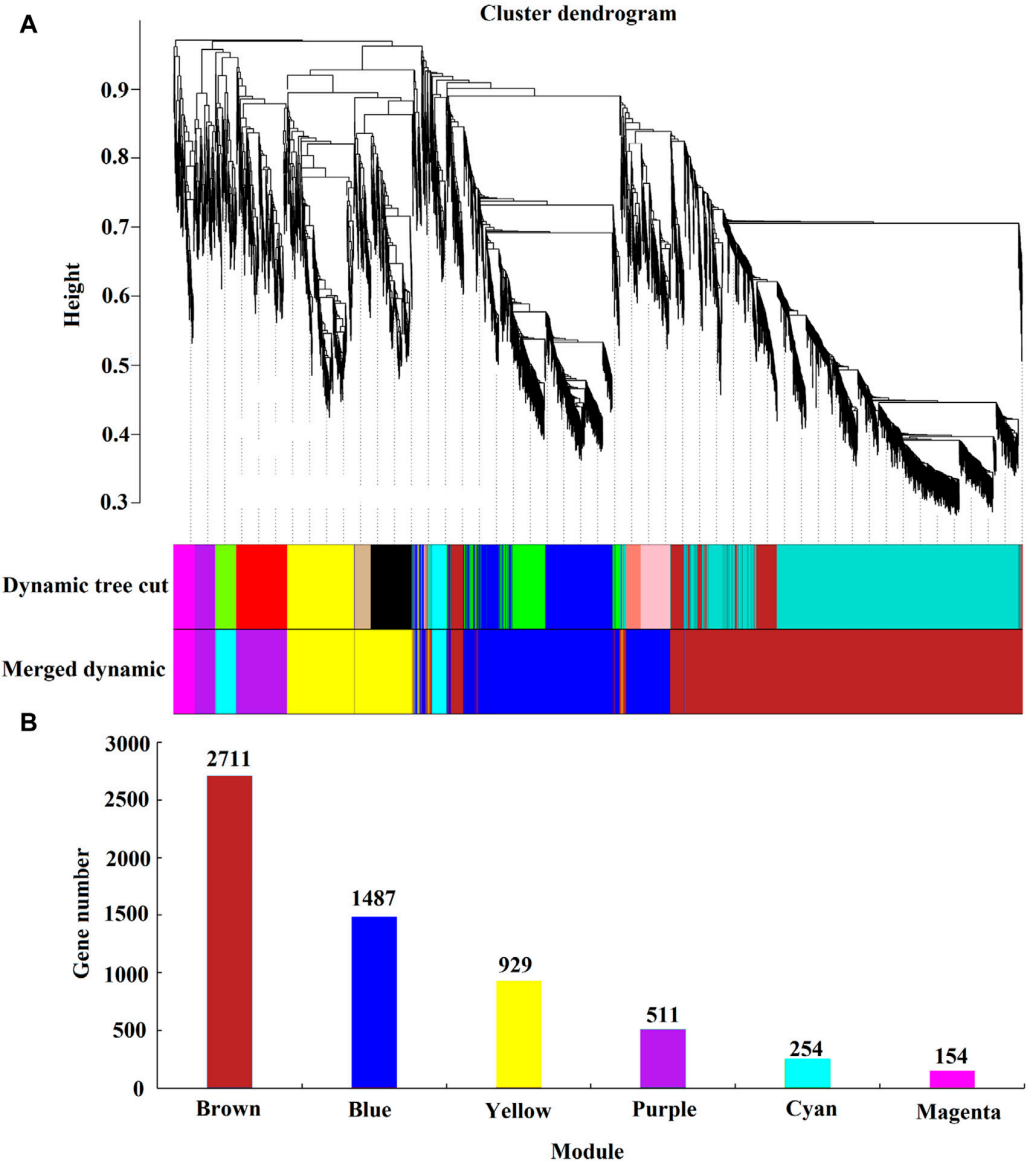
Weighted gene co-expression network analysis. Note: **(A)** Module level clustering diagram; **(B)** Histogram of gene number of each module; Dynamic Tree Cut is the initially acquired module; Merged Dynamic is the Merged module.

Through WGCNA analysis, the clustering results divided differentially expressed genes (FPKM ≥1) with similar expression patterns into six major co-expression modules, as shown in Figure 9. The module with the most genes is the Brown module (2,711 genes), followed by the Cyan module (254 genes), Magenta module (154 genes), Purple module (511 genes), Blue module (1,487 genes), and yellow module (929 genes).

**FIGURE 10:**
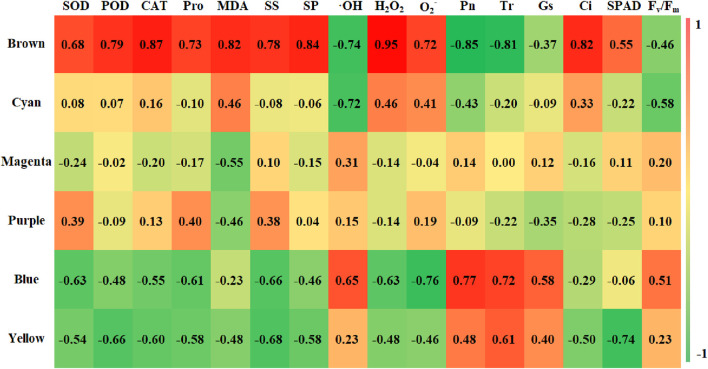
Character correlation diagram. Note: Vertical coordinates for five identified modules, The horizontal coordinates were 16 physiological indexes; the numbers in the graph represent correlation coefficients.

After acquiring the six modules, we proceeded to explore their correlation with the previously identified physiological indicators. The correlation and significance level between the module eigenvalues and physiological indicators were analyzed, and a correlation heatmap was plotted (Figure 10). The results revealed that the Brown module (2,711 genes) had a highly significant positive correlation with CAT, MDA, SP, H_2_O_2_, and Ci, with correlation coefficients of 0.87, 0.82, 0.84, 0.95, and 0.82, respectively, all with a significance level of 0.01. At the same time, SOD, POD, Pro, SS, O_2-_, and SPAD also showed strong correlations with the Brown module, with correlation coefficients of 0.68, 0.79, 0.73, 0.78, 0.72, and 0.55, respectively. The Brown module was found to have a highly significant negative correlation with Pn and Tr, with correlation coefficients of −0.85 and −0.81, both at a significance level of 0.01. The Cyan module was negatively correlated with OH and Fv/Fm, with correlation coefficients of −0.752 and −0.58, and significance levels of 0.05 and 0.13, respectively, while the other 14 indicators had a weaker correlation with the Cyan module. The Blue module was significantly positively correlated with Pn, Tr, and OH, with correlation coefficients of 0.77, 0.72, and 0.65, and significance levels of 0.03, 0.05, and 0.08, respectively. The Yellow module was significantly negatively correlated with POD, SS, and SPAD, with correlation coefficients of −0.66, −0.68, and −0.74, and significance levels of 0.07, 0.06, and 0.04, respectively. The Purple and Magenta modules had weaker correlations with the 16 physiological indicators. Our results underscore the stronger correlations between the Brown module and the 16 physiological indicators, as compared to other modules, prompting us to concentrate our investigation on the genes encompassed within this particular module.

**FIGURE 11:**
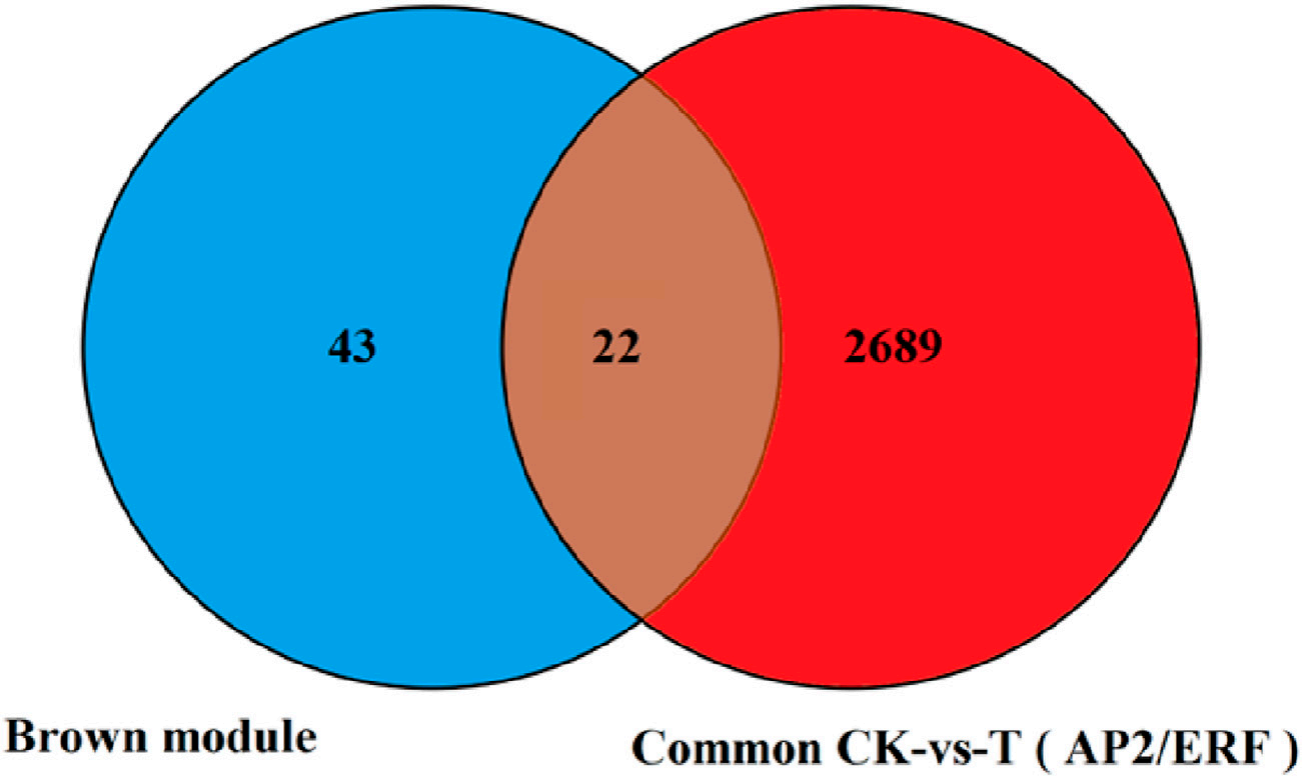
The number of transcription factors differentially expressed in the two combinations was screened by venin map.

In this study, we focused on the Brown module (2,711 genes, including 221 newly discovered genes) that has a strong correlation with physiological indicators. Previous studies have consistently demonstrated that transcription factors occupy a crucial position in regulating plant responses to drought stress. Therefore, we concentrated on the analysis of transcription factors to identify key transcription factors within this module. Our analysis revealed that there are 136 transcription factors in this module, including 36 genes belonging to the AP2/ERF family, 16 to the MYB family, 10 to the bZIP family, 11 to the WRKY family, 6 to the ARF family, and others such as the BTB and CAMTA families. This analysis provides a reference for mining key drought-resistant transcription factors, with the AP2/ERF family being the most abundant. Therefore, based on the 65 differentially expressed AP2/ERF genes screened, we created a Venn diagram with the 2,711 genes in the Brown module identified by the WGCNA co-expression network. As shown in Figure 11, 22 AP2/ERF transcription factors are present not only in the Brown module but also in the significantly differentially expressed AP2/ERF transcription factors. The details of these 22 genes are listed in Table 2. Hence, these genes are deemed as the central transcription factors in quinoa’s drought stress response within the context of this study, and they hold potential for further functional investigations.

**TABLE 2:** Annotation information of 22 core genes.

Gene Id	Gene name	Description
AUR62040582	*CqERF15*	AP2/ERF transcription factor (quinoa)
AUR62018057	*CqERF24*	AP2/ERF transcription factor (quinoa)
AUR62002113	*CqERF28*	AP2/ERF transcription factor (quinoa)
AUR62025525	*CqERF33*	AP2/ERF transcription factor (quinoa)
AUR62028234	*CqERF45*	AP2/ERF transcription factor (quinoa)
AUR62007132	*CqERF53*	AP2/ERF transcription factor (quinoa)
AUR62005714	*CqERF58*	AP2/ERF transcription factor (quinoa)
AUR62017943	*CqERF60*	AP2/ERF transcription factor (quinoa)
AUR62044004	*CqDREB03*	AP2/ERF transcription factor (quinoa)
AUR62009286	*CqDREB05*	AP2/ERF transcription factor (quinoa)
AUR62014452	*CqDREB10*	AP2/ERF transcription factor (quinoa)
AUR62012312	*CqDREB11*	AP2/ERF transcription factor (quinoa)
AUR62022952	*CqDREB12*	AP2/ERF transcription factor (quinoa)
AUR62002024	*CqDREB16*	AP2/ERF transcription factor (quinoa)
AUR62001898	*CqDREB17*	AP2/ERF transcription factor (quinoa)
AUR62003776	*CqDREB27*	AP2/ERF transcription factor (quinoa)
AUR62034546	*CqDREB31*	AP2/ERF transcription factor (quinoa)
AUR62010658	*CqDREB36*	AP2/ERF transcription factor (quinoa)
AUR62010657	*CqDREB37*	AP2/ERF transcription factor (quinoa)
AUR62037338	*CqDREB40*	AP2/ERF transcription factor (quinoa)
AUR62017297	*CqDREB43*	AP2/ERF transcription factor (quinoa)
AUR62009806	*CqDREB49*	AP2/ERF transcription factor (quinoa)

### Plant hormone signal transduction under drought stress

**FIGURE 12:**
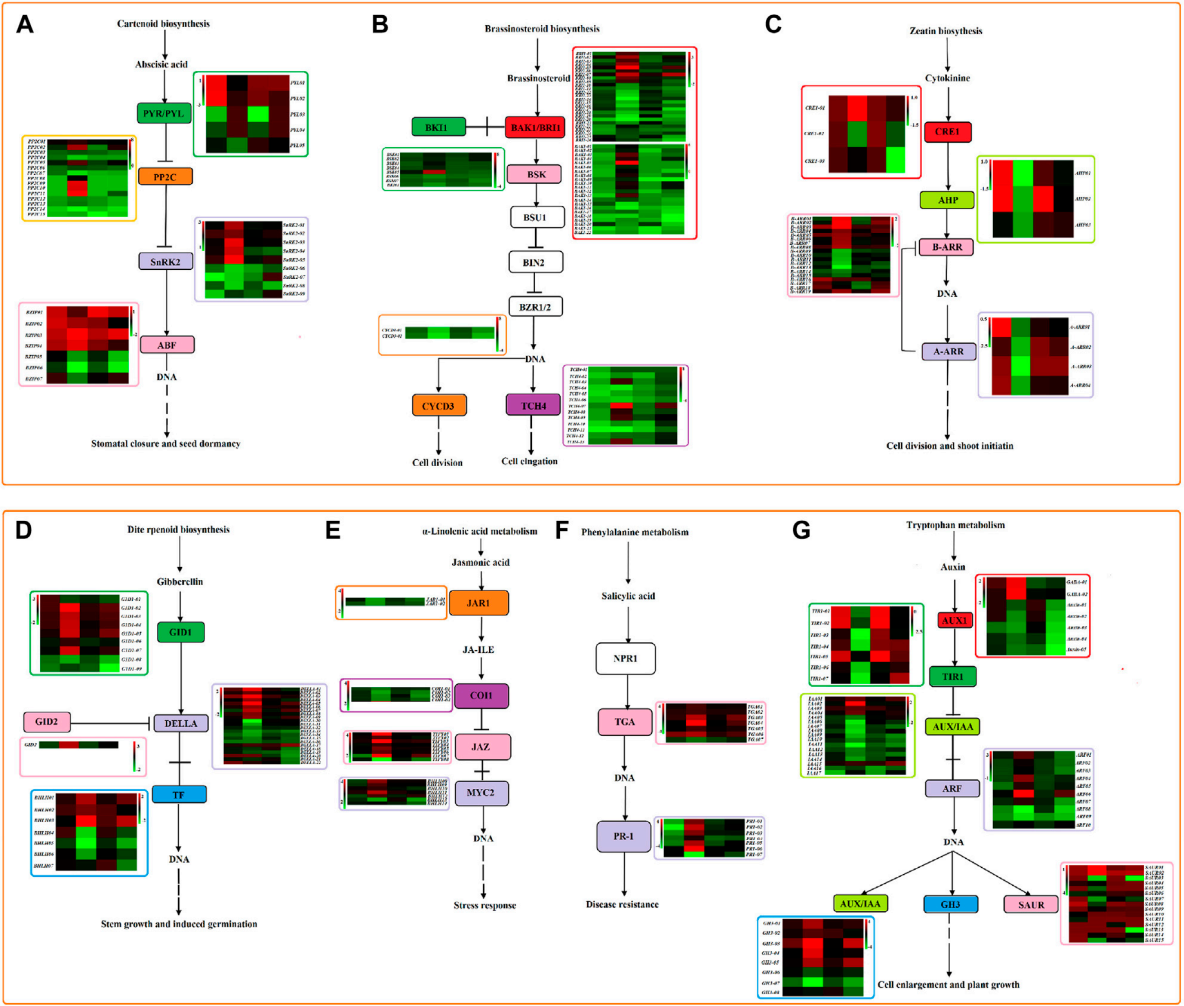
Plant hormone signal transduction pathway under 20% PEG stress based on log2FC value. Note: **(A)**: ABA signaling pathway; **(B)**: BR signaling pathway; **(C)**: CK signaling pathway; **(D)**: GA signaling pathway; **(E)**: JA signaling pathway; **(F)**: SA signaling pathway; **(G)**: IAA signaling pathway. The heat map represents HZ1-C3-vs-T3, HZ1-C9-vs-T9, L1-C3-vs-T3 and L1-C9-vs-T9 from left to right.

At the same time, we detected a large number of differential genes involved in plant hormone signal transduction under quinoa drought stress (Figure 12). In this study, a total of 307 DEGs (Supplementary Table S2) were identified to be involved in auxin (IAA), cytokinin (CK), gibberellin (GA), abscisic acid (ABA), salicylic acid (SA), jasmonic acid (JA), brassinosteroid (BR) metabolism of plant hormone signal transduction. In the ABA signaling pathway, four types of genes (*PYL01*-*PYL05*, *pP2C01*-*PP2C15*, *SnRK2-01*-*SnRK2-05*, *bZIP01*-*bZIP05*) were involved. Among them, four genes (*pP2C01*, *pP2C03*, *pP2C05* and *pP2C06*) were upregulated under two drought stress points of the two genotype materials, and two genes (*PYL03* and *PYL04*) were downregulated on the third day of drought stress in the two genotype materials, but expressed normally on the ninth day. Three genes (*bZIP03*, *bZIP05* and *bZIP06*) were downregulated in both genotypes on the 9th day of drought stress, and *bZIP03* was upregulated, but the three genes were normally expressed on the 3rd day. Gene *SnRK2-02* was upregulated in HZ1 but was normally expressed in L1. In the BR signaling pathway, six categories of genes (*BAK1-01*-*BAK1-22*, *BRI1-01*-*BRI1-26*, *BSK01*-*BSK07*, *BKI01*, *TCH4-01*-*TCH4-13*, *CYCD3-01*-*CYCD3-02*) were involved. Among them, eight genes (*BAK1-10*, *BAK1-11*, *BAK1-04*, *BRI1-02*, *BRI1-05*, *BRI1-07*, *BSK01* and *BSK02*) were upregulated in both genotypes on the 9th day of drought stress. However, *BAK1-10* and *BAK1-11* were not expressed on day 3, and the remaining six genes were normally expressed on day 3. Seven genes (*BRI1-11*, *BRI1-15*, *BRI1-17*-*BRI1-19*, *CYCD3-01* and *CYCD3-02*) were downregulated in both genotypes on day 9 of drought stress, but were normally expressed on day 3. At the same time, one gene BRI1-20 was downregulated under two drought stress points of the two genotype materials. In the CK signaling pathway, four types of genes were involved (*CRE1-01*-*CRE1-03*, *AHP01-AHP03*, *B-ARR01-B-ARR19*, *A-ARR01-A-ARR04*); the *B-ARR02* gene was upregulated in the two genotype materials on the 9th day of drought stress. Four genes were involved in GA signaling pathway (*G1D1-01-G1D1-09*, *GID2*, *DELLA-01-DELLA-22*, *bHLH01-bHLH07*); four genes (*G1D1-08*, *DELLA-13* and *bHLH05*) were normally expressed in the two genotypes on day 3 of drought stress, but were downregulated and *bHLH01* was upregulated on day 9. In the JA signaling pathway, four types of genes were involved (*JAR1-01-JAR1-02*, *COI1-01-COI1-04*, *TIFY01-TIFY08*, *bHLH01-bHLH07*); five genes (*JAR1-01*, *COI1-02-COI1-04* and *BHlH15*) were normally expressed in the two genotypes on day 3 of drought stress, but downregulated on day 9. In the SA signaling pathway, 2 types of genes were involved (*TGA01-TGA07*, *PR1-01-PR1-07*); *TGA03* was normally expressed in the two genotype materials on the 3rd day of drought stress, but was upregulated on the 9th day. In the IAA signaling pathway, 7 types of genes (*GABA-01-GABA-02*; *Auxin-01-Auxin-05*; *TIR1-01-TIR1-07*; *IAA01-IAA17*; *ARF01-ARF10*; *GH3-01-GH3-08*; *SAUR01-SAUR15*); eleven genes (*Auxin-02, TIR1-01-TIR1-03, IAA09, IAA13, ARF08, SAUR07, SAUR15, ARF06* and *GH3-05*) were normally expressed in the two genotypes on the 3rd day of drought stress. Among them, *ARF06* and *GH3-05* were upregulated on the 9th day, and the remaining 9 genes were downregulated on the 9th day.

### Construction of PPI network

**FIGURE 13:**
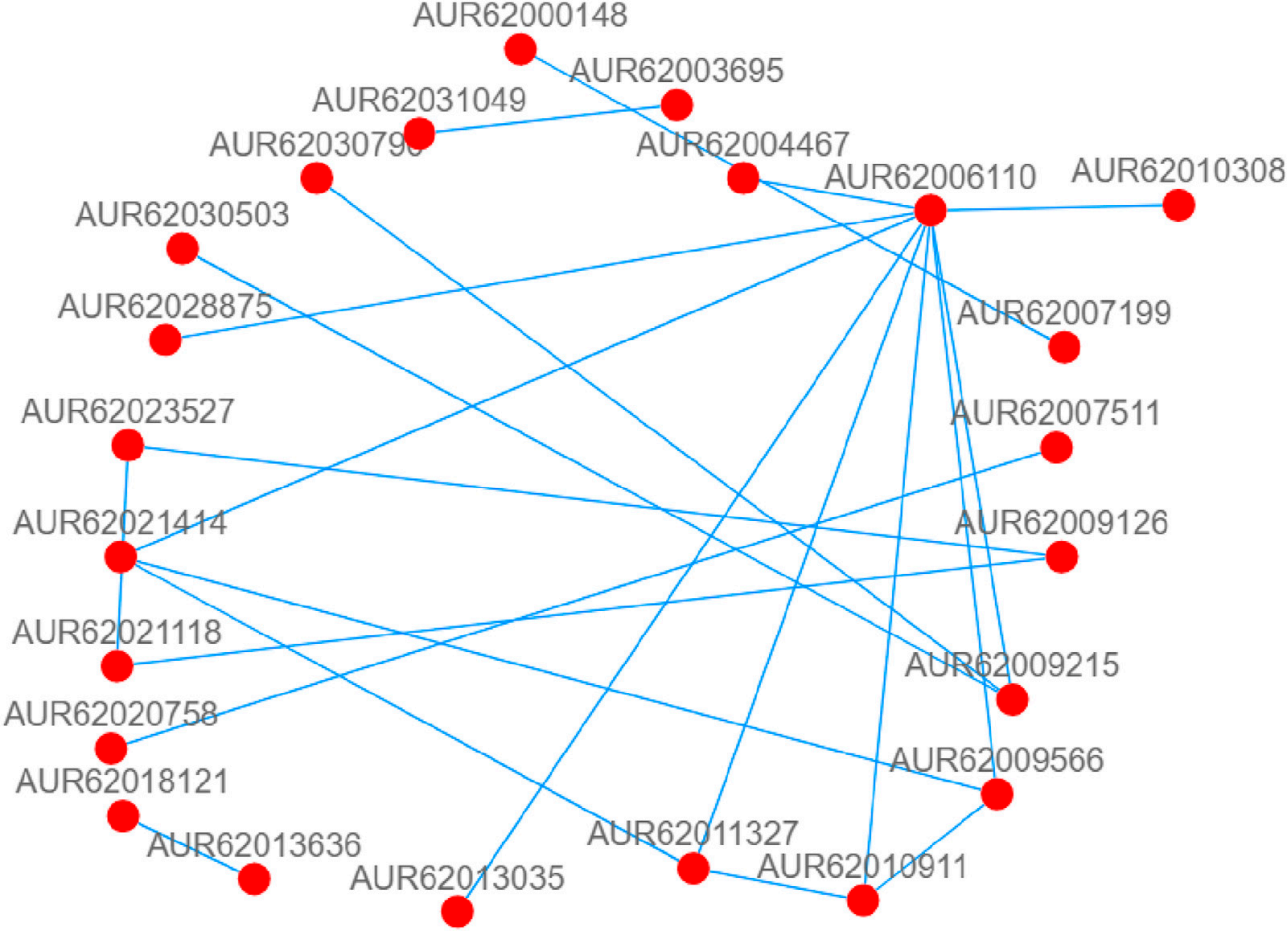
DEGs-related protein-protein interaction (PPI) network of leaf co-responses to drought stress. Note: The nodes are proteins, and the connections are the interactions between them. For visualization purposes, protein-protein interactions with scores below 10 were deleted.

Protein is involved in all cellular activities in plants. Therefore, in this study, we constructed the PPI network by using the protein sequences of 220 differentially expressed genes in L1 and HZ1 under drought stress, to identify key genes. Based on the interaction results, we identified 3 key candidate genes, plasmid lipid-associated protein 14 (PAP14, AUR62006110), and protein phosphate 2C6 (*p*P2C6, AUR62010911 and AUR62021414), implicating these genes to play critical roles in drought stress (Figure 13).

### The differentially expressed genes were verified by qRT-PCR

**FIGURE 14:**
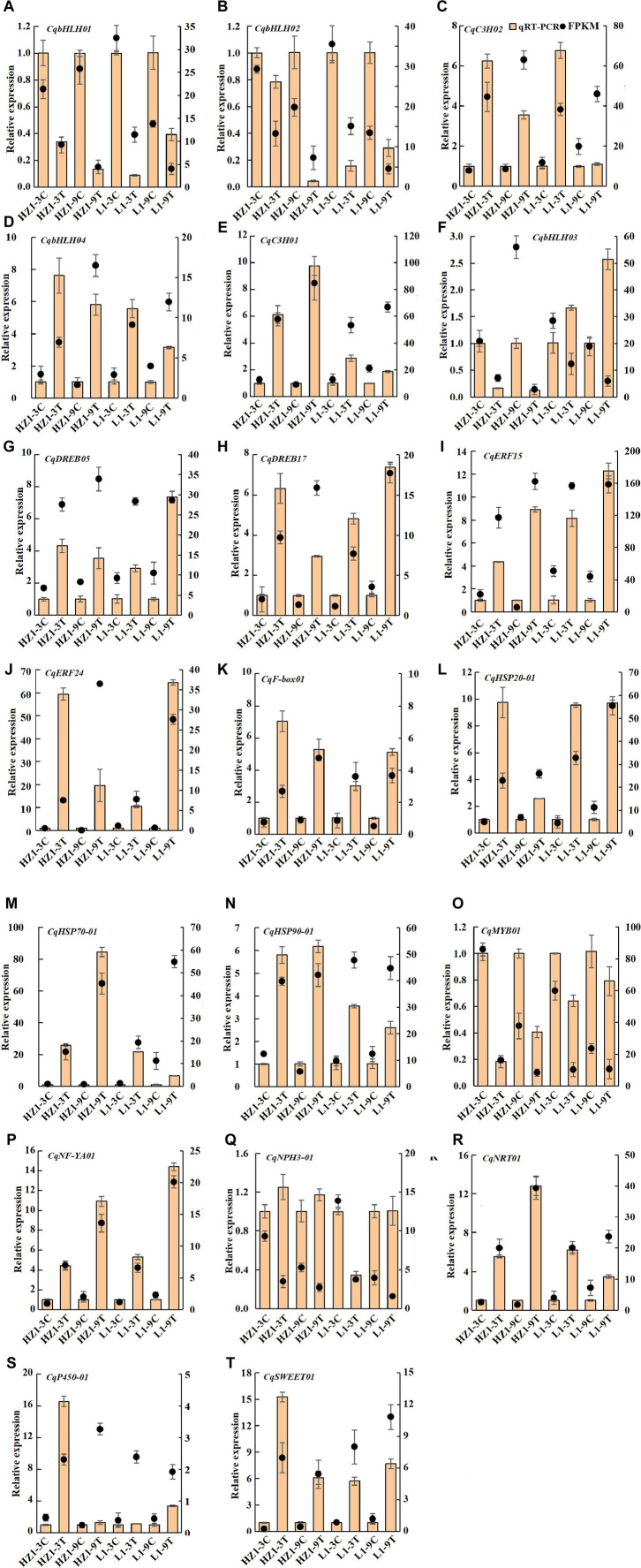
qRT-PCR was used to identify the differentially expressed genes of RNA-seq. Note: Brown columns represent qRT-PCR results, and black dots represent transcriptome data, the right ordinate represents the FPKM value; the data are presented as mean ± SE, and the vertical line represents standard error.

To validate the accuracy of our transcriptome data, we selected 20 differentially expressed genes for qRT-PCR confirmation. 15 genes were upregulated in the transcriptome data, including four AP2/ERF genes, two C3H genes, one gene encoding bHLH, F-box, HSP20, HSP70, Hsp90, NF-YA, NRT1, P450 and SWEET protein respectively, five genes were downregulated in the transcriptome data, including three genes encoding bHLH transcription factor, one gene encoding MYB and one gene encoding NPH3 protein. qRT-PCR results found that two genes (CqbHLH03 and CqNPH3-01) did not match RNA-Seq results, and qRT-PCR results for the remaining 18 genes (90%) were completely consistent with RNA-Seq results (Figure 14).

## Discussion

Photosynthesis serves as the cornerstone for plant growth and development, with water being the paramount factor influencing this vital process. Drought stress will hinder the growth of stamen aperture, reduce rubisco activity and chlorophyll biosynthesis, resulting in a decrease in photosynthesis rate (Parry et al., 2002). The significant decrease of Pn in L1 and HZ1 in this study indicated that continuous drought stress led to significant inhibition of photosynthesis in quinoa. The continuous decrease of Gs indicated that the limitation of CO_2_ absorption caused by stomatal closure was the cause of photosynthesis inhibition. In addition, with the prolongation of drought stress, Ci in L1 and HZ1 began to increase, indicating that metabolic disorders caused by drought became another major limiting factor. In addition, the decrease of HZ1 was greater than that of L1, indicating that HZ1 was more sensitive to drought. Plant leaf chlorophyll concentration assessment is one of the most effective diagnostic tools to study drought tolerance. Plants overcome drought stress by increasing chlorophyll. Therefore, the decrease of chlorophyll concentration due to drought stress is a common phenomenon. The chlorophyll content of plants with strong drought resistance was less affected by drought stress (Sharifi and Mohammad Khani, 2016), which was consistent with the results of this study. We found that the effect of drought stress on HZ1 was significantly greater than that of L1, and the chlorophyll content of both showed a decreasing pattern under drought stress. Part of the reason for the decrease in chlorophyll content due to water shortage could be attributed to drought-induced lipid peroxidation, which arises from the production of reactive oxygen species (ROS), including those derived from molecular oxygen (O_2_) such as O_2_ and H_2_O_2_. Therefore, chlorophyll is also destroyed. Because the green leaves become yellow, the chlorophyll content decreases. It may also be that drought stress hinders the biosynthesis of chlorophyll. Chlorophyll decomposition increased, resulting in decreased chlorophyll content. Chlorophyll fluorescence parameters can also serve as a valuable tool for screening tolerant varieties under abiotic stress conditions, where Fv/Fm serves as a reflection of damage to the PSII center and acts as a suitable indicator for assessing photoinhibition in plants experiencing drought stress. Crop varieties capable of maintaining a high Fv/Fm ratio under drought conditions are considered to be drought-resistant (Zlatev, 2009). In addition, Fv/Fm is positively correlated with the sensitivity of plants to drought (Amor et al., 2018). The Fv/Fm values of the two materials in this study decreased at all time points after drought stress, indicating that the efficiency of light energy conversion and electron transfer activities were significantly inhibited under drought stress, resulting in a decrease in photosynthetic activity, PSII receptor damage, resulting in impaired photosynthesis, decreased electron transport capacity. At the same time, the decrease of Fv/Fm of L1 was far less than that of HZ1, which indicated that the drought resistance of LI was stronger than that of HZ1. Plant cells can also resist the toxicity of reactive oxygen species through protective enzyme systems such as SOD, POD, and CAT, thereby preventing plant cell damage and removing excessive ROS through the system (Keshavarz and Moghadam, 2017). SOD as the primary line of defense against reactive oxygen species, POD can reduce the accumulation of H_2_O_2_, to maintain the integrity of the cell membrane, CAT as an effective enzyme H_2_O_2_ decomposition (Xie et al., 2021). Plants exhibiting drought tolerance accumulate fewer free radicals by effectively maintaining enzymatic activity, and notably, the antioxidant enzyme activity of drought-resistant varieties surpasses that of sensitive varieties, underscoring their enhanced resilience to drought stress (Seyed Ebrahimi et al., 2016). In this study, we found that the SOD, POD and CAT activities of L1 and HZ1 increased under drought stress, and the SOD and POD activities of L1 were significantly higher than those of HZ1 under drought stress, while the superoxide anion production rate and hydrogen peroxide content of L1 were significantly lower than those of HZ1. This indicates that quinoa seedlings can eliminate the excessive accumulation of free radicals by increasing the activity of antioxidant enzymes under drought stress, thereby reducing the damage of drought stress. It was found that the ratio of palisade tissue to spongy tissue increased and leaf density increased with the severity of water deficit, which indicated that the drought resistance of plants was increasing, the relative reduction of the thickness of the developed palisade and spongy tissues helps to improve the water-holding capacity of plants, which indicates that the internal structure of leaves of HZ1 is prone to change significantly after drought stress, the effect of drought stress was much greater than that of L1.

### Transcription factors of quinoa under drought stress

TFs stand as promising candidates for genetic engineering endeavors aimed at cultivating stress-tolerant crops. Their pivotal role as primary regulators of numerous stress response genes underscores their significance in this context. Studies have shown that TFs such as NAC, b-ZIP, AP2/ERF, WRKY, MYB and b-HLH play a key role in plant response to abiotic stresses such as drought by participating in multiple signaling pathways (Wang et al., 2016). Our study revealed that numerous genes encoding transcription factors exhibited differential expression patterns in L1 and HZ1 subsequent to drought stress exposure, including AP2/ERF, b-HLH, NAC, MYB, b-ZIP, WRKY, HD-ZIP and NF-Ys, among which the number of differentially expressed genes encoding AP2/ERF, BHLH and MYB was more. We found that three genes encoding bHLH transcription factors (*AUR62018515-RA*, *AUR62020904-RA* and *AUR62022678-RA*) were downregulated in HZ1-C3-vs-T3, HZ1-C9-vs-T9, L1-C3-vs-T3 and L1-C9-vs-T9, and one gene encoding bHLH transcription factor (*AUR62026299-RA*) was upregulated after drought stress. The bHLH family is one of the largest transcription factors in plants and plays a key role in light signal, hormone signal, wound and drought stress response. Overexpression of wheat *TabHLH39* gene enhances drought resistance, salt tolerance and frost resistance of transgenic Arabidopsis (Zhai et al., 2016). In addition, we found that four genes encoding ERF transcription factors (*AUR62001898-RA*, *AUR62009286-RA*, *AUR62018057-RA* and *AUR62040582-RA*) were upregulated in HZ1-C3-vs-T3, HZ1-C9-vs-T9, L1-C3-vs-T3 and L1-C9-vs-T9. As one of the largest transcription factor families in plants, AP2/ERF plays a key role in plant response to drought stress. Studies have found that the overexpression of soybean *GmDREB1* (Chen et al., 2022), *GmAP2/ERF144* (Wang et al., 2022), *ZmERF21* (Wang et al., 2022) and *ZmEREBP60* (Zhu et al., 2022) improved the drought tolerance of transgenic plants. In our study, 34 and 15 genes were upregulated and downregulated after 9 days of drought stress, respectively. This result indicates that the genes regulated by AP2/ERF TF will change significantly under drought conditions, proving that AP2/ERF TF plays an active role in the regulation of drought stress. In summary, this discussion underscores the pivotal role of TFs in regulating quinoa’s drought response. Notably, various TF families display distinct responses and intricately interact within complex gene regulatory networks, further emphasizing their significance in this process.

### Analysis of starch and sucrose metabolism and phenylpropanoids biosynthesis in response to drought stress

Our study revealed that the majority of differentially expressed genes (DEGs) in quinoa leaves under drought stress were implicated in starch and sucrose metabolic pathways, with glucose metabolism assuming a pivotal role in the stress response mechanism. Notably, glucose metabolism emerged as a crucial determinant of plant adaptability and resilience to diverse environmental stresses. Previous reports have shown that starch and sucrose metabolic pathways exist in many plants under drought stress (Ma et al., 2017; Khan et al., 2019; Hong et al., 2020). Sucrose synthase (SUS) is a key enzyme in sucrose metabolism, because sucrose is important for cell growth. In this study, we identified six DEGs encoding sucrose synthase, of which three genes (*Su-synthase-02*, *Su-synthase-04* and *Su-synthase-06*) were upregulated under drought stress, suggesting that water shortage will lead to increased expression of SUS-related genes in quinoa, which is consistent with previous studies in tobacco (Khan et al., 2019). In addition, we observed 77 DEGs encoding glycoside hydrolases, among which pectinase, polygalacturonase, glucanase, cellulase and xyloglucanase belong to glycoside hydrolases. Most of the genes encoding these enzymes were upregulated during drought stress, suggesting that quinoa may respond to drought stress by hydrolyzing these sugars. At the same time, starch synthase is the largest protein in the starch synthase complex, and its function is to extend long-chain amylopectin in starch synthesis. This study found that a gene encoding starch synthase *G-B-S-synthase-01* was upregulated under drought stress, suggesting that quinoa leaves synthesize excess amylopectin, thereby releasing energy to cope with drought stress. Biosynthesis of phenylpropanoid secondary metabolites is critical for plant response to stress (Deng and Lu, 2017; Sharma et al., 2019). Studies have underscored the significance of phenolic compound accumulation in mitigating the detrimental effects of drought stress on plants. Notably, the primary driver behind this accumulation is the regulation of the phenylpropanoid biosynthetic pathway. Specifically, drought acts as a modulator, influencing the expression of numerous key genes that encode the principal enzymes involved in the phenylpropanoid pathway. Consequently, this upregulation stimulates the biosynthesis of phenolic compounds, contributing to the plant’s resilience against drought (Guo et al., 2018). In this study, 15 genes encoding caffeoyl-CoA3-O-methyltransferase (*CCoAOMT*) -related genes (*O-methyltransferase-01-O-methyltransferase-15*) in the phenylpropanoid metabolic pathway were differentially expressed under drought stress, most of which were upregulated on the 9th day of stress. *AtCCoAOMT1* plays a pivotal role in the biosynthesis of lignin, flavonoids, as well as sinapic acid and malic acid (Do et al., 2007), and studies in peanuts have also shown that related *CCoAOMT* genes are downregulated under drought stress (Zhao et al., 2021). In addition, we found that 58 genes encoding POD (*POD01*-*POD58*) were induced by drought in quinoa, and POD activity was also significantly upregulated under drought stress, indicating that these POD genes were also involved in the response of quinoa to drought stress. The results also confirmed that phenylpropanoid metabolic pathway was activated under drought stress, resulting in a large number of phenolic compounds to deal with the adverse effects of drought.

### Drought stress induced plant hormone signal transduction and MAPK signaling pathway

Drought stress can elicit distinct signal responses in plant organs, encompassing plant hormone signal transduction and the MAPK signaling pathway. Notably, numerous hormone-related pathways within these systems are intimately linked to the plant’s ability to tolerate drought conditions (Gupta et al., 2020). In this study, the auxin inflow vector AUX1-related gene (*Auxin-01-Auxin-05*) required for auxin signal transduction was downregulated under drought stress. Most of the AUX/IAA genes (*IAA01-IAA17*) were downregulated in HZ1 under drought stress. The downregulation of this AUX/IAA gene led to the activation of the downstream ABF gene (*ARF01-ARF10*). The activated ARF gene plays a pivotal role in regulating cell expansion and mediating quinoa’s response to drought stress during its growth cycle. *TaSAUR75* transgenic Arabidopsis showed higher root length and survival rate under drought stress (Guo et al., 2018). In this study, we found that 11 SAUR genes (*SAUR03-SAUR11* and *SAUR15*) were downregulated in the sensitive material HZ1, indicating that they play a pivotal role in modulating plant responses to water deficit. Furthermore, the majority of plants enhance their drought resistance primarily by intricately adjusting stomatal conductance and precisely manipulating the ABA signaling pathway, facilitated by stomatal-specific promoter (Rusconi et al., 2013). The ABA signaling complex PYR/PYL-PP2Cs-SnRK2s was activated in quinoa leaves under drought stress. Four genes encoding *p*P2C (*pP2C01*, *pP2C03*, *pP2C05* and *pP2C06*) were upregulated in both genotypes, five genes encoding SnRK2 (*SnRK2-01*-*SnRK2-05*) were downregulated in HZ1, and two genes encoding PYR/PYL (*PYL03* and *PYL04*) were downregulated in HZ1 and L1, but *PYL01* and *PYL02* were upregulated in HZ1. Studies have shown that overexpression of PYR/PYL inhibits *p*P2C, releases SnRK2, and then activates the downstream target ABF transcription factor (Fujii and Zhu, 2009) ABF binds and activates the promoter of the transcription factor DRE-binding protein 2 A (DRE2A), which plays a key role in drought stress (Kim et al., 2011). The findings of this study revealed that the PYR/PYL pathway was significantly inhibited in HZ1 and L1, whereas the *p*P2Cs pathway exhibited overexpression. JA signaling pathway can alleviate the damage caused by drought stress to plants (Wang et al., 2020). In this study, seven JAZ genes (*TIFY01*-*TIFY07*) and five bHLH genes (*bHLH08*-*bHLH13*) related to JA signal transduction were upregulated on the 9th day of HZ1 drought stress. It was found that OsJAZ1 was a transcriptional regulator of OsbHLH148-related JA signaling pathway, which led to drought tolerance (Seo et al., 2011). This finding further corroborates the role of the bHLH transcription factor as a positive regulator of the JAZ gene. Drought stress additionally induced the upregulation of genes within the SA signaling pathway. In this study, six TGA genes (*TGA01*-*TGA06*) were upregulated on the 9th day of HZ1 drought stress, and the overexpression of *AtTGA4* increased the tolerance to drought stress (Zhong et al., 2015). The findings of this study suggest that the TGA gene significantly enhances the drought tolerance of quinoa *via* the SA signaling pathway. In addition, our study found that most genes in BR, ET and GA signaling pathways were also significantly upregulated or downregulated, indicating that the hormone signaling pathway plays an important role in quinoa response to drought stress. In addition, the MAPK signaling pathway is also induced under PEG stress. The MAPK cascade can convert environmental signals into molecular and cellular responses (Kumar et al., 2020), and the MAPK cascade is related to ABA and ethylene (ET) signals (Jeyaramraja et al., 2018). Our study also found that 85 DEGs were detected in ET and ABA signaling pathways. ABA-activated MAPPPK18 kinase affects stomatal signals under drought stress (Danquah et al., 2015). In this study, ABA upregulated the expression of two MAPKKK18 genes (*MAPKKK-01* and *MAPKKK-02*) on the 9th day of HZ1 material. Therefore, our study postulates that under drought stress, quinoa seedlings protect themselves from drought-induced damage by orchestrating transcriptional changes that involve the induction of plant hormone signal transduction genes and MAPK signal transduction pathway.

## Data Availability

The datasets presented in this study can be found in online repositories. The names of the repository/repositories and accession number(s) can be found in the article/Supplementary Material.
