# Supplementary material for: Molecular and physiological responses of two quinoa genotypes to drought stress
Source: Front Genet. 2024 Aug 9;15:1439046. doi: 10.3389/fgene.2024.1439046 (PMC11341418; doi:10.3389/fgene.2024.1439046)
Supplement: Supplementary file 2 [file Table1.DOCX]

**Table S1** **Summary of RNA Sequencing Results of 24 Quinoa Seedling Leaf Samples**

| #SampleID | Clean reads | GC (%) | Q20 (%) | Q30 (%) | Total read | Mapped reads (%) | Uniq. Map Reads (%) | Multiple Map Reads (%) |
| --- | --- | --- | --- | --- | --- | --- | --- | --- |
| HZ1-C3-1 | 19707233 | 45.28 | 98.22 | 94.57 | 39414466 | 35810078(90.86%) | 32533035(82.54%) | 3277043(8.31%) |
| HZ1-C3-2 | 20666992 | 44.07 | 98.05 | 94.13 | 41333984 | 39802462(96.29%) | 36945802(89.38%) | 2856660(6.91%) |
| HZ1-C3-3 | 20880451 | 44.65 | 98.18 | 94.44 | 41760902 | 39048996(93.51%) | 36535987(87.49%) | 2513009(6.02%) |
| HZ1-C9-1 | 20290590 | 44.8 | 97.01 | 92.24 | 40581180 | 35101948(86.50%) | 28797363(70.96%) | 6304585(15.54%) |
| HZ1-C9-2 | 24289014 | 44.87 | 98.08 | 94.3 | 48578028 | 45785593(94.25%) | 41912314(86.28%) | 3873279(7.97%) |
| HZ1-C9-3 | 24170713 | 44.5 | 97.55 | 93.21 | 48341426 | 45707993(94.55%) | 40851858(84.51%) | "4856135(10.05%) |
| HZ1-T3-1 | 20864629 | 44.12 | 98.22 | 94.58 | 41729258 | 40189120(96.31%) | 37114765(88.94%) | 3074355(7.37%) |
| HZ1-T3-2 | 21030791 | 44.11 | 98.29 | 94.76 | 42061582 | 40613254(96.56%) | 37085229(88.17%) | "3528025(8.39%) |
| HZ1-T3-3 | 19379199 | 44.51 | 98.21 | 94.56 | 38758398 | 36523161(94.23%) | 33667490(86.87%) | 2855671(7.37%) |
| HZ1-T9-1 | 20955571 | 43.92 | 98.22 | 94.59 | 41911142 | 39983879(95.40%) | 37028465(88.35%) | 2955414(7.05%) |
| HZ1-T9-2 | 22031743 | 44.68 | 98.15 | 94.4 | 44063486 | 40497822(91.91%) | 37188641(84.40%) | 3309181(7.51%) |
| HZ1-T9-3 | 20472691 | 43.81 | 98.16 | 94.43 | 40945382 | 38998244(95.24%) | 35505693(86.71%) | 3492551(8.53%) |
| L1-C3-1 | 25722944 | 44.11 | 98.12 | 94.44 | 51445888 | 49132402(95.50%) | 44261446(86.03%) | 4870956(9.47%) |
| L1-C3-2 | 20802071 | 44.15 | 98.13 | 94.42 | 41604142 | 40114535(96.42%) | 36251061(87.13%) | 3863474(9.29%) |
| L1-C3-3 | 20497015 | 44.14 | 98.15 | 94.43 | 40994030 | 39629110(96.67%) | 37162878(90.65%) | 2466232(6.02%) |
| L1-C9-1 | 20171423 | 44.65 | 98.15 | 94.44 | 40342846 | 37445148(92.82%) | "35017103(86.80%) | 2428045(6.02%) |
| L1-C9-2 | 28043885 | 45.08 | 98.35 | 94.95 | 56087770 | 52272722(93.20%) | 44317946(79.02%) | 7954776(14.18%) |
| L1-C9-3 | 21497919 | 44.34 | 98.2 | 94.54 | 42995838 | 41468225(96.45%) | 37150929(86.41%) | 4317296(10.04%) |
| L1-T3-1 | 20571317 | 44.48 | 98.06 | 94.21 | 41142634 | 38793927(94.29%) | 35265477(85.72%) | 3528450(8.58%) |
| L1-T3-2 | 21372385 | 43.83 | 98.11 | 94.31 | 42744770 | 41274598(96.56%) | 38057776(89.03%) | 3216822(7.53%) |
| L1-T3-3 | 22425715 | 44.36 | 98.01 | 94.05 | 44851430 | 42919093(95.69%) | 40611013(90.55%) | 2308080(5.15%) |
| L1-T9-1 | 25431627 | 44.07 | 98.14 | 94.44 | 50863254 | 48797742(95.94%) | 42020532(82.61%) | 6777210(13.32%) |
| L1-T9-2 | 19535975 | 44.13 | 98.15 | 94.44 | 39071950 | 37019353(94.75%) | 33623098(86.05%) | 3396255(8.69%) |
| L1-T9-3 | 20573946 | 43.86 | 98.17 | 94.47 | 41147892 | 39533076(96.08%) | 35994791(87.48%) | 3538285(8.60%) |

Note : ( 1 ) Samples : sample number ; ( 2 ) Clean reads : the total number of pair-end reads in Clean Data ; ( 3 ) GC content : Clean Data GC content, that is, Clean Data G and C two bases in the total base percentage ; ( 4 ) ≥ Q20 % : percentage of bases with a Clean Data quality value greater than or equal to 20. ( 5 ) ≥ Q30 % : percentage of bases with a Clean Data quality value greater than or equal to 30 ; ( 6 ) Total Reads : the number of Clean Reads, single-ended ; ( 7 ) Mapped Reads : the number of Reads aligned to the reference genome and the percentage in Clean Reads ; ( 8 ) Uniq Mapped Reads : compare the number of Reads to the only position of the reference genome and the percentage in Clean Reads ; ( 9 ) Multiple Map Reads : Compare the number of Reads in multiple locations of the reference genome and the percentage of Clean Reads.
