# Supplementary figures and images for: Molecular and physiological responses of two quinoa genotypes to drought stress
Source: Front Genet. 2024 Aug 9;15:1439046. doi: 10.3389/fgene.2024.1439046 (PMC11341418; doi:10.3389/fgene.2024.1439046)

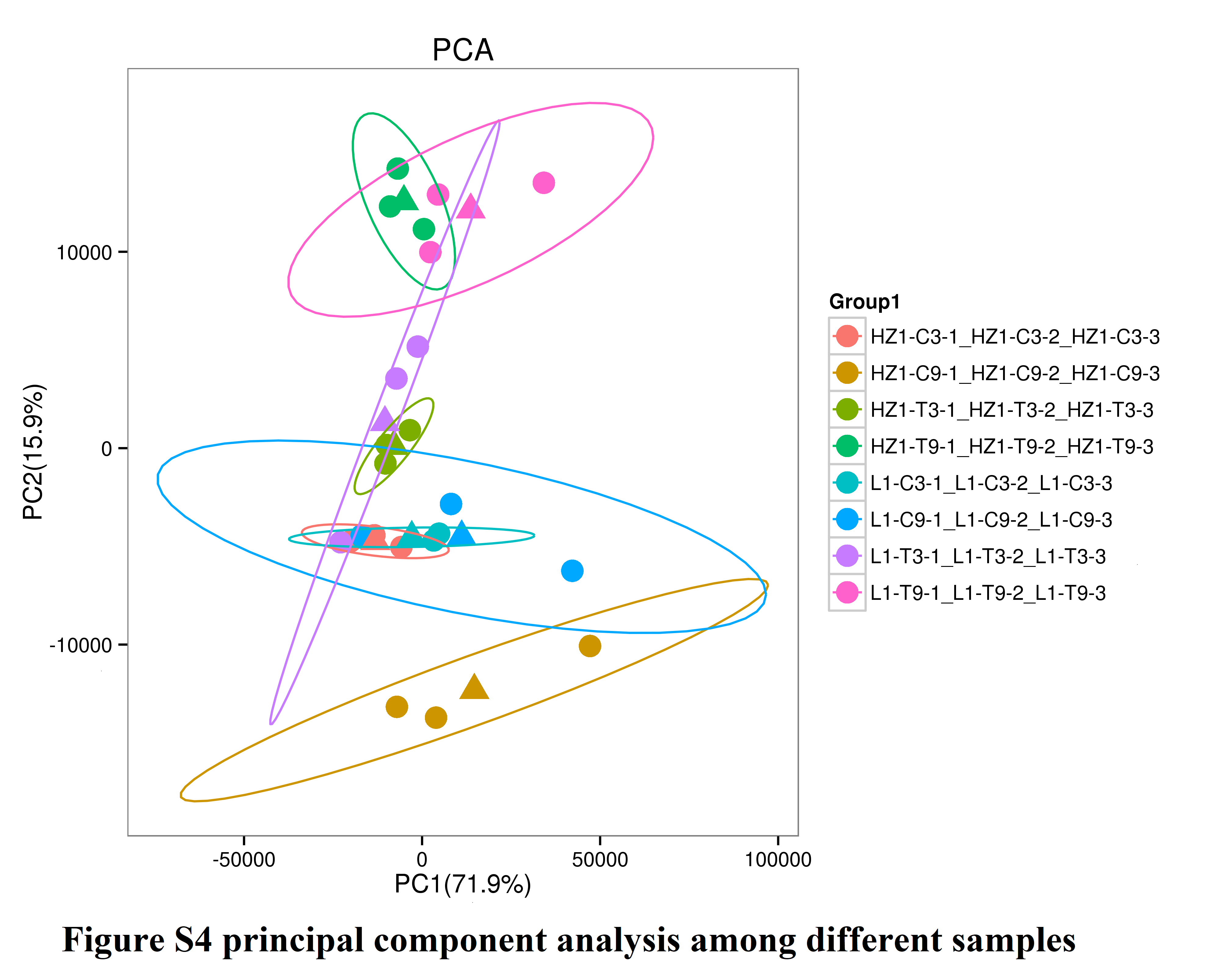

Supplement: Supplementary file 3 [file Image2.TIF]

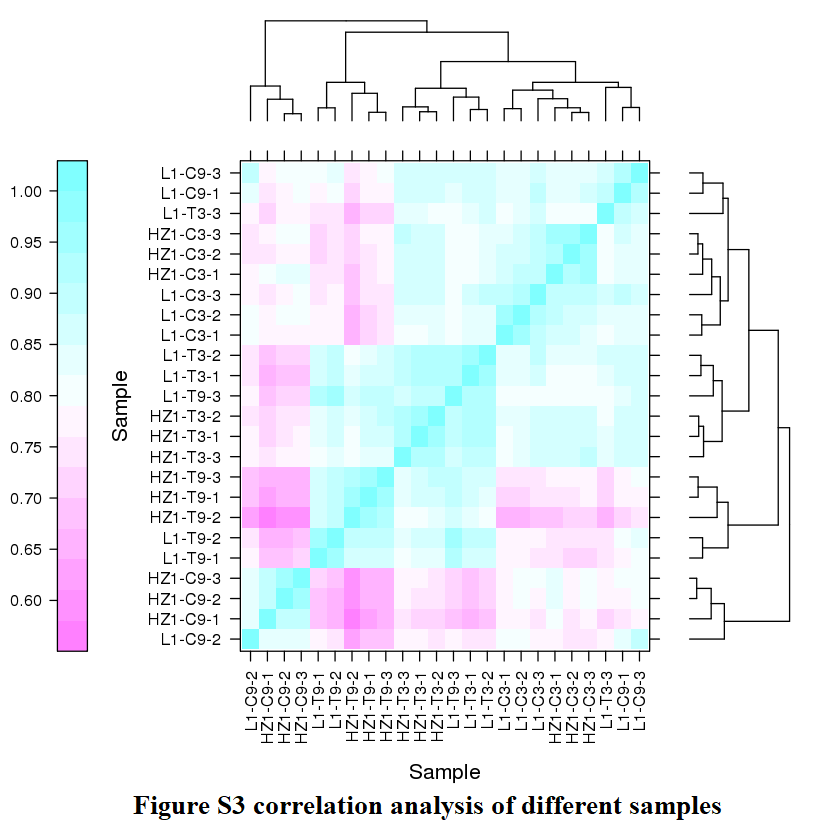

Supplement: Supplementary file 4 [file Image1.TIF]
